# Microsphere‐Modulated Sensing‐in‐Energy Supercapacitor With Self‐Filtering Ultra‐Large Signal Under High‐*g* Shocks

**DOI:** 10.1002/advs.75530

**Published:** 2026-05-06

**Authors:** Zhihao Zheng, Yiqun Wang, Kaiyou Liu, Shancheng Luan, Yajiang Yin, Xiaofeng Wang, Keren Dai, Zheng You

**Affiliations:** ^1^ Department of Precision Instrument Tsinghua University Beijing China; ^2^ School of Mechanical Engineering Nanjing University of Science and Technology Nanjing China

**Keywords:** high‐*g* shock, microsphere‐modulated switch, microsupercapacitor, self‐filtering, Sensing‐in‐Energy

## Abstract

Driven by the “More than Moore” law, the miniaturization and multi‐functional integration of micro‐energy units and micro‐sensors are crucial for next‐generation compact microsystems. To address severe signal oscillation and unstable energy supply under continuous extreme overload (>10 000 *g*), this study proposes a Sensing‐in‐Energy (SiE) microdevice featuring a MEMS movable inertial structure built into a supercapacitor electrolyte cavity. This architecture leverages high‐*g* shock‐driven transient contact between embedded metal microspheres and the electrode to modulate the soft short‐circuit sensing effect. By utilizing electrolyte damping for signal self‐filtering, the device achieves a high‐range (30 000 *g*) and large‐amplitude (450 mV) output with weak oscillation (signal adhesion coefficient reduced by 90.84%). To ensure precise development of the SiE microdevice, a multi‐physics‐driven design method coupling transient fluid‐structure interaction (FSI) and micro‐nano scale rough electrical contact theory was established. This reveals the dynamic mapping laws from mechanical excitation to fluid‐structure coupling interface response and electrochemical output, reducing simulation‐experiment error to within 8%. Furthermore, a high‐precision microsphere embedding process was developed to minimize manufacturing randomness, maintaining signal repeatability error below 10%. This work offers a new paradigm for designing SiE microdevices for extreme environments and lays a technical foundation for the development of future high‐performance heterogeneous microsystems.

## Graphical abstract

To overcome severe signal oscillation under extreme impact conditions, this work presents a Sensing‐in‐Energy microdevice which integrates a MEMS inertial structure directly within a supercapacitor electrolyte cavity. Utilizing shock‐driven transient contact and electrolyte damping for signal self‐filtering, it achieves weak‐oscillation, large‐amplitude, and high‐range sensing. This architecture establishes a robust paradigm for next‐generation heterogeneous microsystems.

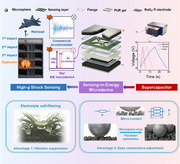

## 1 Introduction

Driven by the “More than Moore” law, the miniaturization [1, 2, 3, 4, 5] and multi‐functional integration [6, 7, 8, 9, 10, 11, 12, 13, 14, 15, 16] of micro‐energy units and micro‐sensors have become the core pathway for developing compact microsystems, aiming to overcome the performance bottlenecks of specialized equipment in extreme environments such as deep space exploration [17, 18], implantable medical care [19, 20, 21, 22], and high dynamic shock [23, 24, 25]. Taking the onboard control system as an example, microsystems are required to synchronously achieve high‐fidelity, oscillation‐free sensing and uninterrupted energy supply under ultra‐high overload (>10 000 *g*) environments. However, extreme mechanical shock poses a fatal threat to the reliability of both traditional sensors and power devices [26]: the former is prone to severe signal attenuation and oscillation that interfere with layer identification, while the latter faces challenges of electrical connection failure and degraded energy retention capability. In particular, when sensors and capacitors adopt a discrete architecture, the degradation of signal quality and failure risks of both components superimpose, which can easily lead to functional failures in the decision‐making and execution of the onboard control system. Therefore, breaking physical boundaries through functional integration to achieve the intrinsic fusion of sensing and energy supply has become the key to enhancing the reliable operation of onboard control systems in extreme environments [27, 28].

To address the aforementioned bottlenecks, the design concept of “Sensing in Energy (SiE)” has emerged, aiming to realize the fusion of sensing and energy supply functions within a single microdevice by embedding sensing functional materials—such as piezoelectric, piezoresistive, or piezocapacitive materials—or sensing functional structures, like deformable electrodes, into micro‐energy storage devices such as supercapacitors [29, 30, 31]. Recently, Ma et al. successfully developed planar micro‐supercapacitors based on 2D heterostructures, achieving a fully flexible and self‐sustained intelligent microelectronic system capable of NH_3_ detection [32]; furthermore, they elucidated the unique multi‐directional ion transport mechanisms in planar microelectrodes and prospectively proposed the concept of intelligent structural microbatteries with simultaneous electrochemical and mechanical load‐bearing capabilities [33, 34], which profoundly propels the paradigm shift of microscale energy storage toward multifunctional adaptive subsystems and outlines a visionary roadmap for next‐generation AI‐driven microelectronics. Zhao et al. proposed bio‐inspired gradient carbon nanotube arrays [35], and Wei et al. introduced hierarchically porous bismuthene‐graphene aerogels [36], which not only endowed the devices with excellent electrochemical energy storage properties but also realized highly sensitive pressure and strain sensing. Li et al. introduced all‐textile materials featuring ion‐electron interfaces [37], while Wang et al. proposed AgNW/MnO_2_ hybrid conductive networks [38]; both approaches effectively resolved the challenges of signal stability and high sensitivity under tensile deformation. Furthermore, addressing miniaturization demands, Zheng et al. developed MXene‐derived microsupercapacitors [39] and further verified the feasibility of integrating high‐performance energy storage units with sensing systems through a high‐voltage window design. These advances have laid a solid foundation for the development of next‐generation self‐powered flexible electronic skins and smart wearable platforms. In contrast, extreme mechanical conditions involving high‐*g* shock sensing present unique challenges, as the aforementioned sensing mechanisms based on continuous deformation are prone to severe signal oscillation caused by inertial delay and structural resonance [40]. To address this challenge, Huang et al. proposed a soft short‐circuit mechanism [41] based on the phase change of separator materials [42] to realize an integrated sensing‐energy supercapacitor, providing a new perspective for simultaneous sensing and energy supply in high‐*g* shock environments. Wang et al. proposed a structural soft short‐circuit mechanism by embedding a planar cantilever beam into a supercapacitor [43]. By utilizing electrolyte damping to modulate the shock deformation of the cantilever, a weak‐oscillation signal output was achieved. Furthermore, microsystem integration and experimental verification of penetrating multi‐layer targets were successfully implemented within onboard control systems.

Overall, the structural soft short‐circuit mechanism is currently the optimal technical path for realizing high‐*g* shock SiE microdevices. However, under this architecture, there are still many issues to be addressed regarding the design and optimization of the integrated microdevice. First, in previous studies, the cantilever mass and the energy storage electrode adopted a planar contact interface, which is unfavorable for the enhancement of the sensing signal amplitude and pulse width. Second, the actual sensing characteristics of this type of integrated device are relatively sensitive to the machining and assembly precision of the movable structure, and there is still a lack of a high‐precision, standardized, and facile manufacturing process.

To address the aforementioned problems, this study proposes a soft short‐circuit SiE architecture featuring a microsphere‐embedded MEMS movable beam structure. Compared with previous studies, the curved contact interface between the embedded metal microspheres and the energy storage electrode can achieve the concentration and enhanced modulation of contact stress, thereby amplifying the soft short‐circuit capacitive effect under high‐*g* shock and realizing the enhancement of sensing signal amplitude and pulse width. On this basis, a fluid‐structure interaction (FSI) – electrical contact – electrochemical coupling modeling and simulation system for the microsphere‐embedded SiE microdevice is proposed. The influences of microsphere material, radius, and array pattern on the sensing signal are analyzed, and a multi‐physics simulation‐driven design optimization framework based on “array dominance, material optimization, and radius regulation” is constructed. Furthermore, a manufacturing process for the integrated photolithography of the movable sensitive beam structure and microsphere positioning pits is proposed. Leveraging the precision advantage of photolithography positioning and the convenience of microsphere electroforming, high‐precision and facile manufacturing of the SiE microdevice is realized. Finally, comprehensive high‐*g* shock response performance testing and characterization were conducted on the fabricated SiE microdevice, verifying its performance advantages in terms of signal amplitude and adhesion coefficient. This provides a novel core microdevice combining high‐fidelity sensing and stable energy supply for the reliable operation of onboard control systems in complex high‐*g* shock environments.

## 2 Results and Discussion

### 2.1 Concept and Mechanism

**FIGURE 1:**
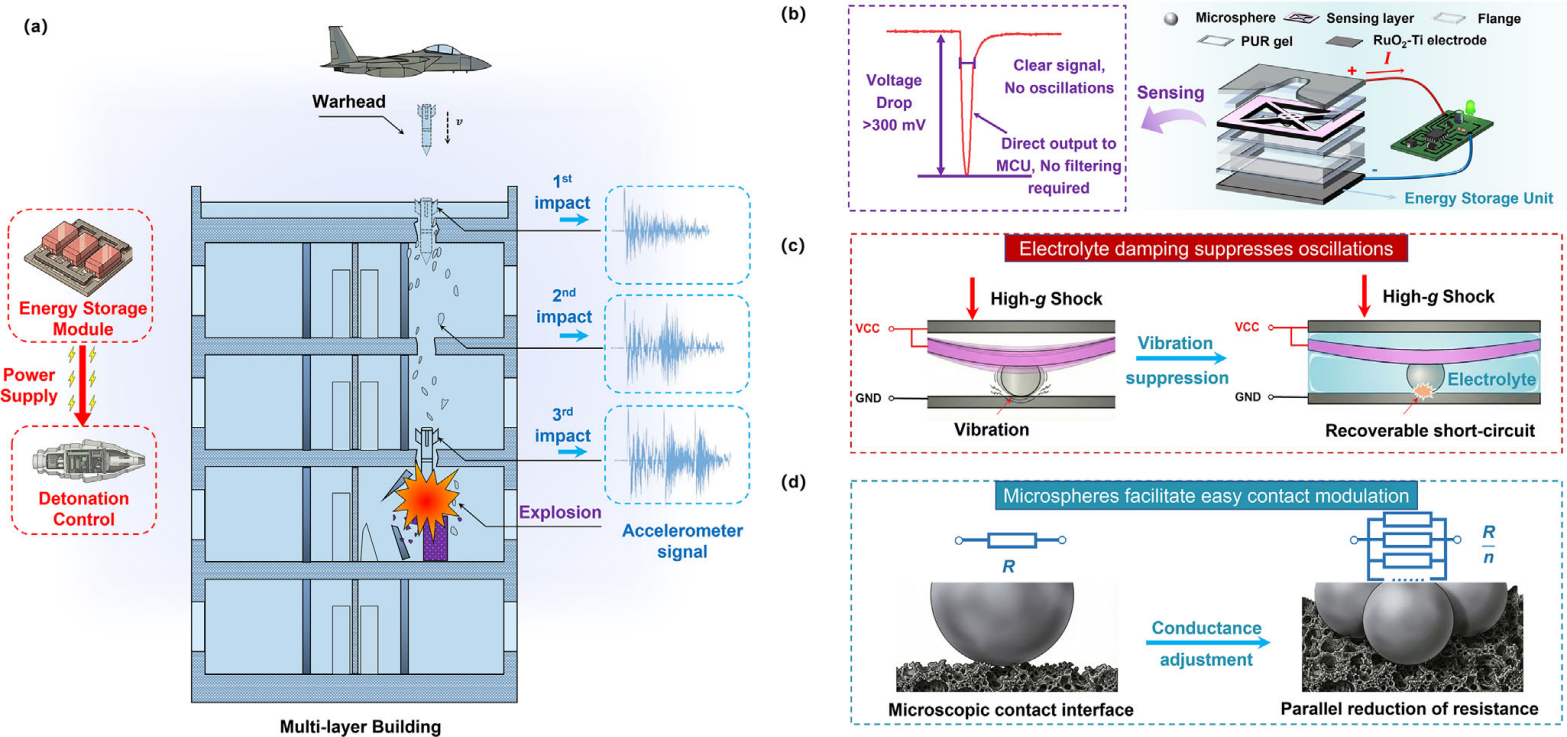
Conceptual design, structural composition, and working mechanism of the SiE microdevice. (a) The SiE microdevice needs to meet the requirements for accurate shock sensing during high‐speed continuous penetration and stable energy supply under high‐*g* shock; (b) Structural composition of the microsphere‐based SiE microdevice: Ruthenium oxide porous electrodes serve for energy storage and powering, while the microsphere‐embedded cantilever inertial switch enables shock sensing, featuring high‐amplitude and oscillation‐free shock response signals; (c) Shock sensing mechanism of the microsphere‐based SiE microdevice: Oscillation suppression is achieved via electrolyte damping, and signal sensing is realized through a single “contact‐separation” transient short‐circuit; (d) Advantages of the novel microsphere‐based SiE microdevice in shock sensing signals: The microsphere array structure facilitates microscopic tuning of the contact interface and precise threshold setting.

As shown in Figure 1a, when intelligent munitions strike multi‐story buildings, they rely on the acceleration sensor of the onboard control system to perceive the number of penetrated building layers, thereby achieving precise lethality with intelligent detonation at a specific layer. However, challenges are posed by the interlayer adhesion of sensor signals caused by oscillation and the shock failure of the sensor power supply. This study proposes a SiE microdevice, the core concept of which lies in integrating a microsphere‐based inertial switch inside a supercapacitor, with the conceptual design shown in Figure 1b. This design aims to maintain the stable energy supply characteristic of the supercapacitor (see Note S1) while utilizing the inertial response of the built‐in cantilever‐microsphere structure and fluid damping for oscillation suppression, thereby overcoming the signal adhesion problem under high‐speed continuous shocks and achieving accurate layer counting sensing.

Figure 1b details the structural configuration of the microdevice. Porous ruthenium oxide is adopted as the electrode material placed at the top and bottom, forming a supercapacitor with sulfuric acid electrolyte to achieve high energy density storage. A four‐arm cantilever beam is constructed using flame retardant 4 (FR‐4) laminate, which possesses both excellent mechanical strength and flexibility, as the substrate. This serves as a carrier for welding a titanium microsphere, acting as the sensitive intermediate layer of the stacked structure. FR‐4 flanges are used to precisely regulate the layer spacing, PUR structural adhesive is utilized for interface sealing, and epoxy resin is supplemented for overall potting, significantly enhancing the structural integrity and robustness of the microdevice under extreme overload.

Based on the aforementioned “energy storage layer – sensing layer – energy storage layer” sandwich structure, Figure 1c illustrates the force‐electric coupling sensing mechanism of the SiE microdevice. The cantilever sensitive layer embedded with the microsphere maintains an electrical connection with the positive electrode of the supercapacitor. Under high‐*g* shock, the inertial force drives the sensitive layer to move toward and contact the bottom electrode. After the shock, the elastic force of the cantilever drives the sensitive layer to recover to the initial position. Upon the occurrence of a contact short‐circuit, a substantial influx of electrons flows through the external circuit into the positive electrode of the SiE microdevice, disrupting the local charge equilibrium. At this moment, a massive amount of hydrogen ions (H+) from the adjacent electrolyte is adsorbed by the electrode particles, entering the electric double layer (EDL) to form solid‐phase hydrogen. Concurrently, a fraction of the H+ undergoes redox reactions on the surface of the electrode particles. This rapid consumption leads to a precipitous drop in the H+ concentration near the positive electrode, which consequently lowers the electrode potential, manifesting as the macroscopic voltage drop process [44, 45]. Once the contact short‐circuit terminates, the diffusion of hydrogen ions facilitates a gradual charge restoration. The rebound of the H+ concentration in the vicinity of the positive electrode leads to an increase in the electrode potential and the compensation of the EDL charge, thereby forming the voltage recovery process [46, 47]. This dynamic sequence constitutes the fundamental signal generation mechanism of the SiE microdevice under high‐*g* impact.

Unlike the continuous mechanical oscillation and “multiple contact” phenomena common in traditional inertial switches under high‐*g* environments, the proposed microdevice utilizes the significant fluid damping effect of the internal electrolyte to effectively suppress parasitic oscillations after contact. Specifically, due to the vacuum injection process utilized in the SiE microdevice, the electrolyte cavity is almost entirely filled with an incompressible liquid. Within this highly confined, micrometer‐scale hermetic space, the downward motion of the microsphere and the four‐beam structure under impact mechanically forces the underlying liquid to be squeezed and displaced laterally through extremely narrow micro‐gaps. This mandatory fluid displacement generates immense squeeze‐film damping and viscous dissipation. Such intense fluidic damping highly efficiently dissipates the kinetic energy of the moving microstructures; furthermore, a higher dynamic viscosity of the fluid yields a correspondingly stronger damping effect (The relationship between electrolyte viscosity and microsphere dynamics is detailed in Note S2). This mechanism fundamentally suppresses any potential post‐impact mechanical oscillations [48, 49, 50]. This “mechanical filtering” mechanism ensures a single, deterministic “contact‐separation” transient response between the microsphere and the bottom electrode, which consequently generates a distinct “drop‐recovery” single‐pulse fluctuation in the supercapacitor terminal voltage. This voltage characteristic serves as the shock sensing signal. A typical response signal is shown in Figure 1b. Its voltage drop can reach hundreds of millivolts. The sensing signal features a high signal‐to‐noise ratio and no parasitic oscillation, allowing direct acquisition by the MCU without pre‐filtering circuits. After the SiE microdevice is stacked with supercapacitor single cells to form a high‐voltage module, the rated voltage has an appropriate redundancy design compared to the load requirement of the onboard control system. Therefore, the sensing voltage drop at the moment of high‐*g* shock does not affect the stable power supply of the module to the control system.

**FIGURE 2:**
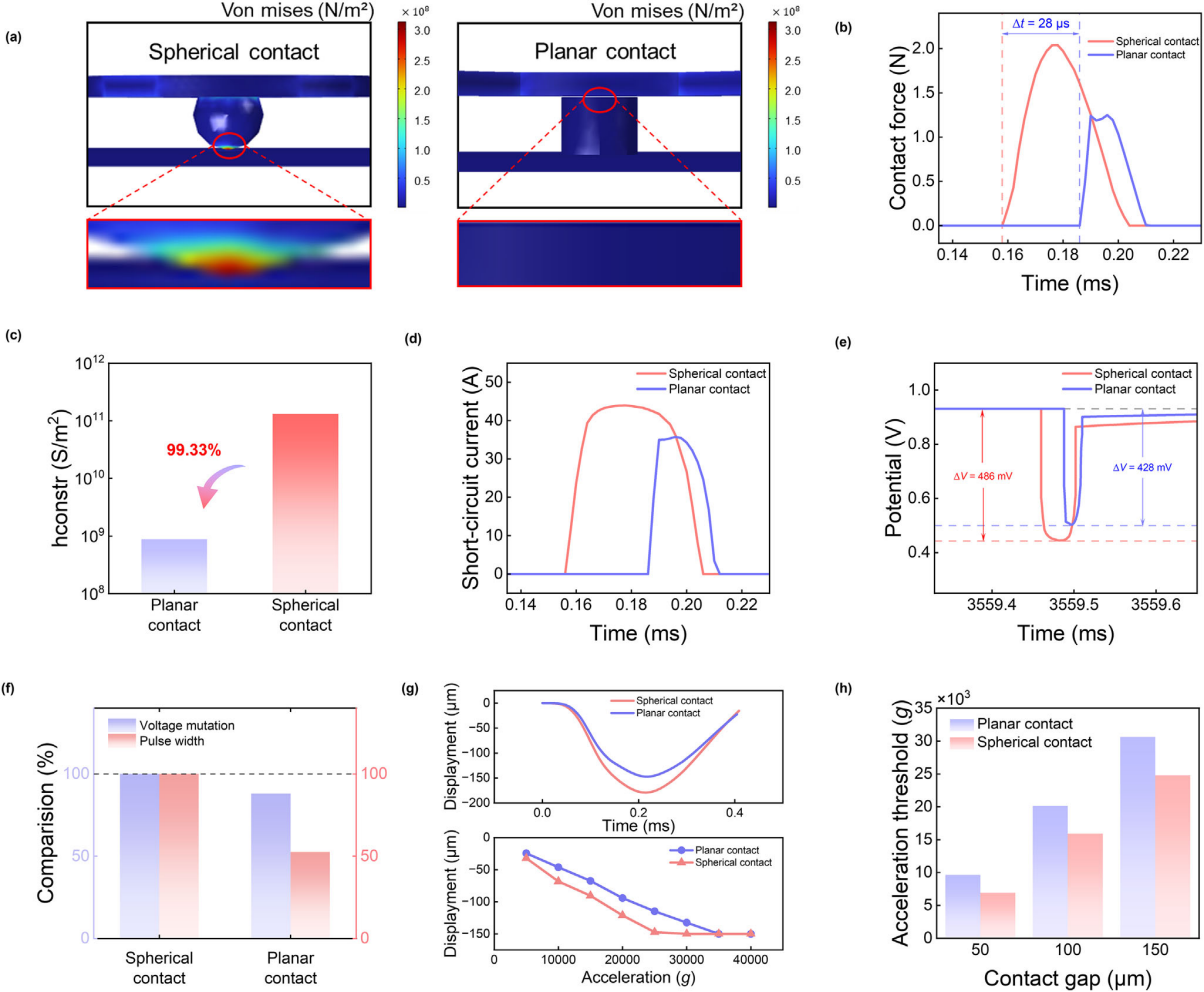
Comparative performance analysis of microsphere‐based and planar inertial switches based on multi‐physics coupling simulation. (a) Stress distribution between contact pairs for microsphere structural contact and planar structural contact under identical high‐*g* shock; (b) Time‐domain response curves of contact pressure for microsphere structural contact and planar structural contact under identical high‐*g* shock; (c) Contact conductivity of microsphere structural contact is significantly superior to that of planar structural contact under identical high‐*g* shock; (d) Time‐domain response of short‐circuit current for microsphere structural contact and planar structural contact under identical high‐*g* shock; (e) Time‐domain response of shock response voltage for microsphere structural contact and planar structural contact under identical high‐*g* shock; (f) Shock sensing signal of microsphere structural contact is characterized by high amplitude and large pulse width compared to planar contact; (g) Time‐domain response curves of displacement for microsphere and planar structures under identical high‐*g* shock, and displacement capabilities of microsphere and planar structures under shocks with different *g*‐values; (h) Under different contact gaps, the shock acceleration threshold for initiating contact in the microsphere structure is lower than that in the planar structure.

Compared with the conventional planar contact structure, the SiE microdevice based on microsphere contact exhibits significant advantages (Figure 1d): the spherical contact possesses lower contact resistance and higher signal amplitude (see the discussion in Figure 2c,e). Furthermore, the microsphere array structure provides additional design degrees of freedom, enabling the tuning of the micromechanical behavior of the contact interface and the precise setting of the acceleration threshold by modifying the microsphere arrangement. To deeply elucidate the physical mechanism of the aforementioned performance enhancement, Figure 2 will provide a quantitative comparative analysis of the microsphere structure and the planar structure from the two dimensions of contact mechanics and electrical response based on a multi‐physics coupling model, revealing the intrinsic mechanism of their performance differences (Detailed assumptions of the multi‐physics model are provided in Note S3).

Based on the simulation results of the multi‐physics coupling model, under high‐*g* shock, attributed to the unique geometric characteristics of spherical contact, the localized concentrated stress at the contact interface of the microsphere structure is significantly higher than the distributed stress of the planar structure (Figure 2a). Signal simulations of both structures under identical high‐*g* shock indicate that spherical contact not only generates greater contact pressure but also possesses a distinct advantage in response speed (Figure 2b).

In terms of electrical performance, although the spherical contact is smaller than the planar contact from the perspective of macroscopic contact area (see Figure S3), in reality, any solid surface is rough at the microscopic scale, consisting of countless undulating asperities. When two conductive surfaces come into contact, physical contact occurs only at the tips of these asperities, establishing actual conductive channels. The load is not uniformly distributed across the entire interface but is concentrated on these discrete contact spots. To quantitatively characterize the modulation mechanism of contact pressure on interfacial conductance, this study adopts the Cooper–Mikic–Yovanovich (CMY) plastic contact correlation model. This model first establishes the constitutive relation between the dimensionless contact pressure and the fractional real contact area. Under the contact mechanism dominated by plastic deformation, the ratio of the real contact area *A*_r_ to the nominal contact area *A*_a_ is determined by the macroscopic contact pressure *p* and the material microhardness *H*_c_, as shown in Equation (1).

(1) $$\frac{A_{r}}{A_{a}}\;\approx \;\frac{p}{H_{c}}$$

As indicated by Figure 2b, the spherical contact possesses greater contact pressure compared to the planar contact, thus possessing a larger real contact area. The CMY theory further introduces surface topography parameters (surface roughness average height σ_asp_ and surface roughness average slope *m*_asp_) to construct a general correlation for contact conductance *h*_c_, as shown in Equation (2).

(2) $$h_{c}=1.25\sigma_{\mathrm{contact}}\frac{m_{\mathrm{asp}}}{\sigma_{\mathrm{asp}}}{\left(\frac{p}{H_{c}}\right)}^{0.95}$$

where σ_contact_ is the harmonic mean conductivity of the contact interface. From Equations (1) and (2), it can be seen that an increase in contact pressure *p* leads to a near‐linear expansion of the real contact area *A*_r_, thereby driving a significant enhancement of the contact conductance *h*_c_. Verification through multi‐physics simulation reveals that the planar contact conductivity is only 0.67% of that of the spherical contact (Figure 2c). To analyze the variation of current during contact, the composition of the current is first discussed. Conduction current dominates during the contact process, while the magnitude of displacement current is negligible (Figure S4). Combining the intrinsic resistance of each component, the evolution curves of contact current/short‐circuit current were obtained (Figure 2d), with the microsphere structure exhibiting a higher current peak.

Substituting the aforementioned parameters into the equivalent circuit model (Figure S5) for the solution, the shock response voltage curve of the SiE microdevice is obtained, as shown in Figure 2e. The response voltage amplitude of the microsphere structure reaches 486 mV, outperforming the 428 mV of the planar structure. To comprehensively evaluate the sensing performance, Figure 2f compares the signal characteristics of the two: the signal amplitude generated by the microsphere structure is 1.136 times that of the planar contact, and the pulse width is 1.906 times larger. These signal characteristics of high amplitude and wide pulse width significantly improve the signal‐to‐noise ratio, making it more adaptable to the layer counting identification algorithm of the onboard control system.

Beyond electrical characteristics, Figure 2g, h further elucidates the superior dynamic response and threshold characteristics of the microsphere structure. Focusing on the fluid‐structure interaction process in the electrolyte environment, the displacement‐time curves of the key nodes of the inertial mass block (the bottom vertex of the microsphere and the center point of the planar beam) were extracted (Figure 2g). Under identical shock loads, the microsphere structure exhibits higher displacement sensitivity by virtue of the introduced added mass and the low fluid damping brought by the streamlined structure. Furthermore, parametric sweep results show (Figure 2h) that under different electrode spacings, the triggering threshold of the microsphere structure is only 70%–85% of that of the planar structure. This characteristic endows the microdevice with a broader threshold tunable range, offering greater potential for threshold design targeted at specific application scenarios.

### 2.2 Parametric Analysis and Optimization Strategy

**FIGURE 3:**
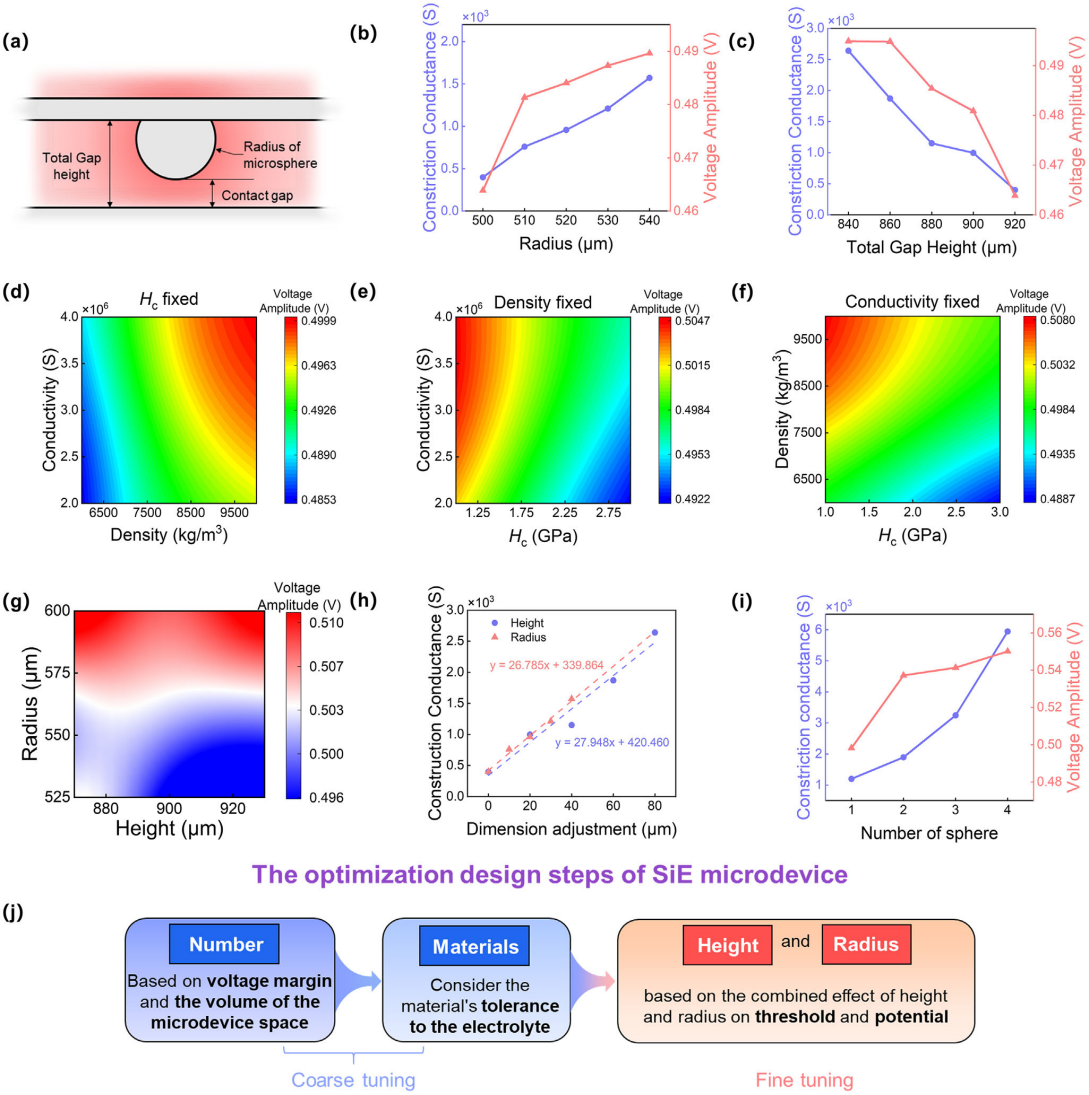
Structural parameter modulation of sensing performance and optimization design strategy for the microsphere‐based SiE microdevice. (a) Key optimization targets for the SiE microdevice: microsphere material properties, microsphere radius, and total gap height between the sensing layer and bottom electrode; (b) Influence of microsphere radius on contact conductance and response signal amplitude under high‐*g* shock; (c) Influence of total gap height on contact conductance and response signal amplitude under high‐*g* shock; (d) Relationship between response signal amplitude and microsphere conductivity and density under 30 000 *g* shock; (e) Relationship between response signal amplitude and microsphere conductivity and microhardness under 30 000 *g* shock; (f) Relationship between response signal amplitude and microsphere density and microhardness under 30 000 *g* shock; (g) Relationship between response signal amplitude and microsphere radius and total gap height under 30 000 *g* shock; (h) Influence of equal‐scale variations in microsphere radius and total gap height on contact conductance under high‐*g* shock; (i) Influence of microsphere number on contact conductance and response signal amplitude under high‐*g* shock; (j) Overall optimization design workflow for the SiE microdevice.

The comparative analysis above mechanistically confirms that the microsphere structure possesses significant advantages over the traditional planar structure in terms of contact mechanics and electrical response, specifically manifesting as higher response voltage amplitude and superior threshold tunability. However, to meet the identification and decision‐making requirements of onboard control systems in complex shock environments, the microdevice must be capable of outputting raw signals with a high signal‐to‐noise ratio and large amplitude to avoid the need for additional signal filtering and amplification circuits. In light of this, this section further employs multi‐physics simulation methods to systematically investigate the influence laws of key geometric parameters of the sensitive layer on shock response characteristics, thereby establishing an optimal structural design strategy for the SiE microdevice (Figure 3).

As shown in Figure 2 previously, the analysis based on contact mechanics indicates that the establishment of contact conductance essentially depends on the evolution of the real contact area of the interface, which is directly controlled by the transient contact pressure at the moment of the microsphere impacting the electrode. This contact pressure is determined by the dynamic response of the microsphere sensitive structure in the fluid environment. Under high‐*g* shock, the motion state of the microsphere is governed by the dynamic balance of inertial force, fluid damping force, structural elastic restoring force, gravity, and buoyancy (Figure S6). Obviously, parameters such as microsphere radius (*R*), total sensitive layer – electrode gap height (*H*), microsphere material, and the number of microsphere arrays (*n*) have clear relationships with the aforementioned mechanical terms. These parameters modulate electrical contact and response signal output by altering the system's mass, damping, and total displacement. The geometric definitions of some parameters are shown in Figure 3a.

The regulatory effects of the microsphere radius and total gap height are the most predictable. Theoretical analysis indicates that, with other conditions held constant, an increase in microsphere radius increases the inertial mass and contact area, but simultaneously intensifies the fluid damping effect. In regions far from the electrode, the fluid damping force is proportional to the microsphere radius *R*. However, in the near‐electrode contact region, due to the squeeze‐film effect, the sensitivity of fluid damping force to *R* increases significantly, being approximately proportional to *R* ^2^ (Note S8). Through multi‐physics simulation, the influence of microsphere radius on contact conductance and response voltage amplitude is shown in Figure 3b. Under controlled variable conditions, both contact conductance (*h*_c_) and response voltage amplitude show an increasing trend with the increase of microsphere radius *R*. It is evident that the enhancement of inertial kinetic energy and contact area dominates the shock process, effectively compensating for the energy loss caused by fluid damping.

The total gap height is a critical kinematic parameter determining the microsphere's travel stroke and impact kinetic energy at the moment of contact. As shown in Figure 3c, when the contact gap is relatively large (*H* >  910 µm), the contact conductance and response voltage amplitude exhibit a monotonic decay trend with increasing gap. The physical mechanism behind this phenomenon lies in stroke‐dependent energy dissipation: under large gap conditions, the extended travel stroke of the microsphere leads to a significant increase in the work done by fluid damping, thereby dissipating more kinetic energy. Furthermore, the action time of the inertial force is relatively short and has already ceased by the time of contact. Considering both factors, this results in a significant reduction in momentum and contact pressure at the instant of contact. It is worth noting that when the gap is in an extremely small range (*H* <  880 µm), the microsphere makes contact during the early stage of the high‐*g* shock (i.e., while the microsphere is still in the high acceleration phase). At this point, the intense inertial load causes the contact behavior to exhibit complex non‐monotonic characteristics (see Figure S7 for details). The competitive mechanism of fluid‐structure interaction under such small gaps will be analyzed in depth later in Figure 3g.

Combining the analysis from Figure 2c, microsphere material parameters have a significant impact on electrical contact behavior. Specifically, density (ρ) influences the FSI dynamic process by altering the microsphere's inertial force; electrical conductivity (σ) directly determines the harmonic mean conductivity of the contact interface; and microhardness (*H*_c_) controls the real contact area between the microsphere and the electrode. Figure 3d–f further quantitatively reveals the influence mechanisms of the aforementioned material properties on sensing performance: the response voltage amplitude shows a clear positive correlation with density and conductivity, but decreases monotonically with increasing microhardness *H*_c_. These results suggest that adopting microsphere materials with high density, high conductivity, and low hardness can substantially enhance the response voltage amplitude, thereby significantly improving the sensor's signal‐to‐noise ratio.

While studying the regulatory effect of total gap height in Figure 3c, a complex influence on contact conductance and response voltage amplitude was observed. To investigate the regulation mechanisms of gap and radius more deeply, a joint parametric sweep of microsphere radius and total gap height was conducted in this study, as shown in Figure 3g. The analysis results indicate that in the high‐inertia region with a large microsphere radius (*R* > 575 µm), the response voltage exhibits a significant “high‐low‐high” non‐monotonic evolution characteristic as the contact gap increases. Specifically, the system achieves a high voltage response when the total gap height is in either a small (*H* < 880 µm) or large (*H* > 910 µm) range, whereas a distinct response depression appears in the medium gap region (around 900 µm). This is attributed to the temporal coupling mechanism between the microsphere motion time (*t*_m_) and the external shock pulse width (τ). In the small gap zone (*t*_m_  <  τ), the microsphere impact occurs near the phase of peak acceleration (see Figure S8), where contact behavior is dominated by the peak inertial force, manifesting as a high response amplitude. As the gap increases and enters the “phase mismatch zone,” the impact moment gradually moves away from the peak inertial acceleration moment. The decay of the instantaneous driving force combined with insufficient kinetic energy accumulation leads to the response dropping to a valley value. In the large gap zone, the kinetic energy accumulation effect brought by the long acceleration stroke takes dominance; the massive impact kinetic energy causes severe deformation of the interface, resulting in a significant rebound in the response voltage amplitude. This result demonstrates that avoiding the energy attenuation zone under small gap conditions is key to maximizing the signal amplitude of the microdevice.

To quantify the influence of the aforementioned geometric dimensions and determine the priority of microsphere radius and total gap height regulation, Figure 3h compares the evolution of conductance under equal dimensional increments. The results show that the regulation of conductivity by both microsphere radius and total gap height is highly linear, and the fitting slopes are extremely close (*K*_R_  =  26.785 S/µm and *K*_H _=  27.948 S/µm, respectively, with a difference of only about 4%). This implies that within the current design domain, the microsphere scale effect and the gap dynamic effect possess equivalent sensitivity in enhancing contact performance. Furthermore, Figure 3i explores the enhancement effect of multi‐microsphere parallel arrays. As the number of microspheres increases, the contact conductance shows progressive growth, and the response voltage is significantly improved (specifically, when the number of microspheres increases from 1 to 4, the response voltage amplitude increases by approximately 80 mV). This indicates that signal sensing performance can be significantly enhanced through array‐based design.

Based on the above analysis, a hierarchical optimization design strategy is proposed: (1) Array Dominance: Determine the number of microsphere arrays based on the rated voltage margin of the integrated system and the spatial volume of the microdevice; (2) Material Selection: Select the optimal material system based on the material's tolerance to the electrolyte; (3) Radius‐Gap Joint Regulation: Based on the “high‐low‐high” evolution map, precisely match the total gap height and radius to avoid the “phase mismatch zone” and meet threshold requirements. This workflow ensures the performance optimization of the SiE microdevice under complex high‐*g* shock.

### 2.3 Fabrication Process and Experimental Validation

**FIGURE 4:**
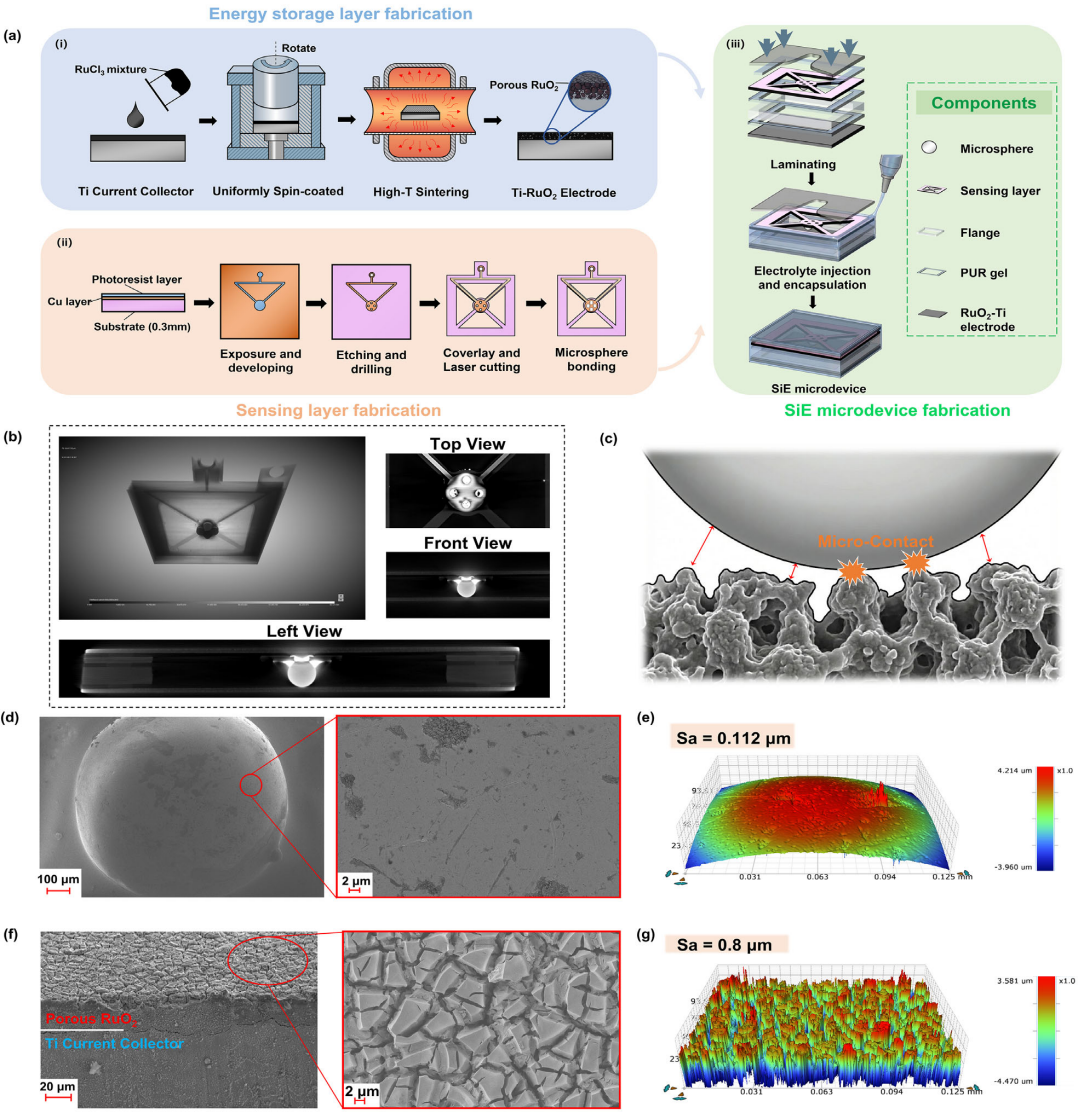
Fabrication process and microstructural characterization of the microsphere‐based SiE microdevice. (a) Fabrication process flow of the SiE microdevice; (b) X‐ray images showing the overall structure, internal microsphere, and stacked edges of the SiE microdevice; (c) Microscopic contact schematic between the microsphere and the ruthenium oxide electrode. (d) SEM image of the entire microsphere; (e) 3D surface morphology of the microsphere; (f) SEM image of the ruthenium oxide electrode; (g) 3D surface morphology of the ruthenium oxide electrode.

Based on the optimization design discussed above, this study further constructed a fabrication process for the SiE microdevice. The feasibility of this fabrication scheme was verified through multi‐scale macroscopic and microscopic characterization of the microdevice, as shown in Figure 4.

Figure 4 comprehensively illustrates the entire process from the microdevice fabrication workflow to macroscopic structural verification, and finally to microscopic morphology characterization. Figure 4a is a schematic diagram of the fabrication process flow for the SiE microdevice, demonstrating the step‐by‐step manufacturing and assembly strategy. Panel (i) depicts the preparation of the energy storage electrode: a mixed precursor of ruthenium chloride was uniformly coated onto a titanium current collector using a spin‐coating process, followed by high‐temperature sintering to form a porous ruthenium oxide electrode layer. Panel (ii) shows the micro‐machining of the sensing layer: precise cantilever beams and microsphere positioning structures were constructed on a flexible substrate via photolithography, developing, etching, and laser cutting technologies. Panel (iii) demonstrates the final lamination assembly process, where the microspheres, sensing layer, flange structure, PUR structural adhesive, and electrode layers were stacked in sequence to construct the complete SiE microdevice. To clearly characterize the overall internal and external structure of the microdevice, x‐ray transmission imaging was performed (Figure 4b). Non‐destructive testing of the packaged microdevice was conducted using x‐ray tomography technology. The top view, front view, and left view clearly present the internal topological structure of the microdevice, confirming that the functional microsphere achieved precise self‐alignment within the cavity center. Furthermore, the multi‐layer stacked structure exhibited tight edge bonding without misalignment or structural collapse, verifying the reliability of the packaging process and structural integrity.

Figure 4c vividly depicts the microscopic contact interface between the microsphere and the rough porous ruthenium oxide electrode surface. This schematic highlights the concept of “micro‐contact spots,” meaning that under high‐*g* shock, the microsphere makes physical contact with the micro‐nano scale peak‐and‐valley structures on the electrode surface, establishing conductive pathways. This characteristic of contact area varying with pressure serves as the physical basis for the microdevice to achieve high‐sensitivity mechano‐electrical conversion.

To deeply investigate the material basis and contact behavior of the microscopic interface, Figure 4d–g displays the microscopic morphology characterization results of the microsphere and the bottom porous electrode, respectively. Figure 4d,e presents the surface morphology characterization of the conductive microsphere. The scanning electron microscope (SEM) image in Figure 4d shows that the microsphere possesses an extremely high degree of sphericity overall, and its locally magnified view reveals a relatively smooth and dense surface without obvious crack defects. The 3D surface morphology reconstruction in Figure 4e further quantifies this feature, measuring an arithmetical mean surface roughness (*S*_a_) of approximately 0.112 µm. Figure 4f,g shows the microstructural characterization of the porous ruthenium oxide electrode. The SEM image in Figure 4f displays the cross‐sectional and surface features of the electrode. The cross‐sectional view clearly shows the bonding interface between the porous ruthenium oxide layer and the Ti current collector substrate; the surface view presents a characteristic cracked microscopic texture, a structure that significantly increases the effective specific surface area of the electrode. The 3D morphology map in Figure 4g reveals rich high‐frequency roughness features on the electrode surface, with an *S*_a_ of approximately 0.8 µm. The densely distributed micro‐asperities on its surface provide a large number of potential contact sites for the microsphere, which is conducive to significantly improving contact conductance.

**FIGURE 5:**
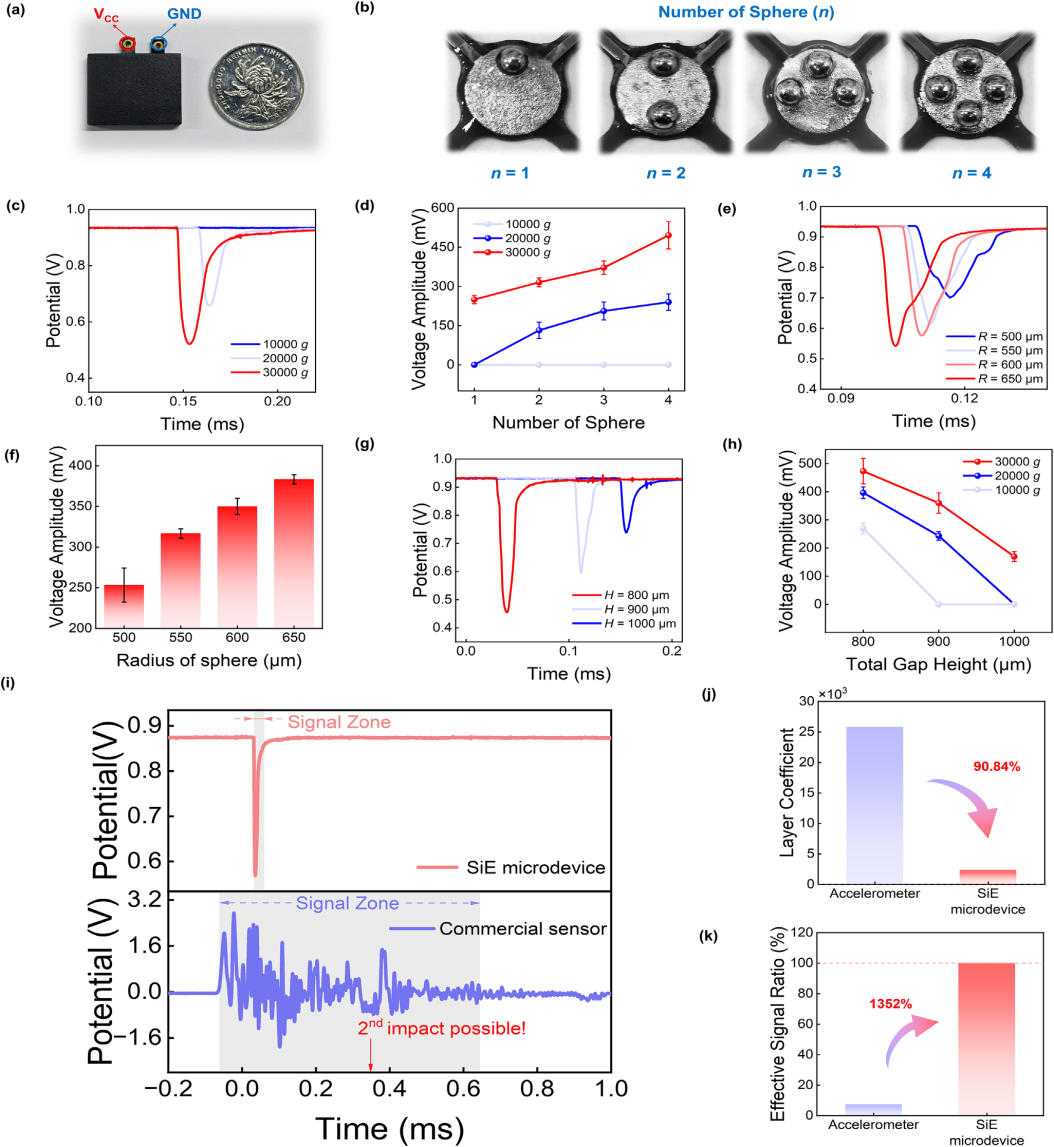
Experimental validation of sensing performance of the microsphere‐based SiE microdevice. (a) Optical image of the potted SiE microdevice. (b) Local optical images of sensing layers with different numbers of microspheres. (c) Time‐domain voltage response curves of the SiE microdevice under 10 000, 20 000, and 30 000 *g* shocks. (d) Measured response voltages and error bars of SiE microdevices with different microsphere numbers under 10 000, 20 000, and 30 000 *g* shocks. (e) Time‐domain voltage response curves of SiE microdevices with different microsphere radius under identical *g*‐value shocks. (f) Measured response voltages and error bars of SiE microdevices with different microsphere radius under 10 000, 20 000, and 30 000 *g* shocks. (g) Time‐domain voltage response curves of SiE microdevices with different total gap heights under identical *g*‐value shocks. (h) Measured response voltages and error bars of SiE microdevices with different total gap heights under 10 000, 20 000, and 30 000 *g* shocks. (i) Time‐domain response signals of the SiE microdevice and a commercial piezoelectric accelerometer under a single high‐*g* shock. (j) Significant reduction in signal adhesion coefficient of the SiE microdevice compared to the commercial accelerometer. (k) Significant improvement in the effective signal ratio of the SiE microdevice compared to the commercial accelerometer.

Benefiting from the fabrication process of the SiE microdevice described previously, this study achieved the stable preparation of the microdevice, laying the experimental foundation for verifying the multi‐physics regulation laws revealed by the simulation in Figure 3. Figure 5a presents a photograph of the packaged SiE microdevice. The microdevice features compact dimensions (comparable to a coin) and integrates a standardized power supply (*V*_CC_) and ground (GND) interfaces. To reveal the microscopic configuration of its internal sensitive unit, Figure 5b provides optical micrographs under different array scales, clearly displaying the structural evolution characteristics from a single microsphere (*n* = 1) to a four‐microsphere array (*n* = 4).

To systematically evaluate the sensing characteristics of the microdevice and verify the regulation mechanism of structural parameters, this study conducted multiple sets of Machete hammer (see Figure S9) high‐*g* shock experiments (Figure 5c–h). Figure 5c depicts the transient voltage response curves of the microdevice under different shock loads ranging from 10 000 to 30 000 *g*; the results show that the response voltage amplitude increases significantly with the increase of the shock *g*‐value, while the response time decreases. Figure 5d further statistically analyzes the influence of the number of microspheres on sensing performance. The results indicate that as the number of parallel microspheres increases, the response voltage amplitude of the microdevice exhibits a progressive growth trend, attributed to the multiplication effect of parallel conductive pathways. Figure 5e,f reveals the modulation law of the microsphere radius on the output signal. The consistency between the time‐domain waveforms (Figure 5e) and the statistical data (Figure 5f) demonstrates that the response voltage increases monotonically with the increase of the microsphere radius (*R* ranges from 500 to 650 µm). This phenomenon strongly corroborates the aforementioned theoretical mechanism: under specific gap conditions, a larger microsphere radius implies higher inertial mass and contact pressure, thereby significantly enhancing contact conductance. Figure 5g,h explores the influence of the contact gap. Comparing the test results under different total gap heights (*H* =  800,  900,  and 1000 µm) with the same microsphere radius, it is observed that the peak response voltage attenuates significantly as the total gap height increases. This suggests that under the current testing conditions, a smaller gap is more conducive to achieving high‐sensitivity detection. The above experimental results provide a solid experimental basis for the optimal design of the microdevice's structural parameters.

To verify the consistency between simulation and experiment, this study compared the experimental test curve under a 30 000 *g* shock with the finite element simulation results, as shown in Figure S10. The two agree well in terms of waveform characteristics and amplitude, powerfully verifying the accuracy of the established theoretical model. The experimentally obtained response voltage amplitude is slightly higher than the simulation prediction (by no more than 8%). This is primarily attributed to non‐ideal factors in the manufacturing process: trace residual air that is difficult to completely eliminate during the vacuum injection process alters the local fluid‐structure interaction dynamics, leading to a slight increase in the experimental response amplitude (as shown in Figure S11).

The performance comparison between the SiE microdevice and a commercial sensor is shown in Figure 5i,j,k. Figure 5i records the output signal comparison between the SiE microdevice and a commercial piezoelectric accelerometer under identical high‐*g* conditions. After the shock, the commercial sensor exhibits severe oscillation and secondary shock signals, which interfere with the interpretation of the real signal and would seriously affect the layer counting identification of subsequent shocks. In contrast, the output signal of the SiE microdevice is clean and oscillation‐free. The quantitative analysis in Figure 5j,k shows that the signal adhesion coefficient (the definition is shown in Note S14) of the SiE microdevice is 2364.58, which is 90.84% lower than that of the commercial accelerometer (25 817.30). Meanwhile, its effective signal ratio is 13.52 times that of the commercial accelerometer. This fully demonstrates its high signal fidelity and anti‐adhesion characteristics in high‐*g* environments. Additionally, we systematically reviewed and compared the test results from recent literature on high‐*g* shock sensors. The comparison primarily focuses on three core performance metrics: (1) Self‐powered capability; (2) Typical output voltage amplitude; (3) Oscillation ratio (defined here as the amplitude ratio of the second characteristic peak to the first characteristic main peak in the shock sensing signal). The results are presented in Table 1, from which it can be clearly seen that compared to the traditional piezoresistive and capacitive accelerometers reported in existing high‐*g* literature, the SiE microdevice proposed in this study exhibits a distinct advantage in signal response amplitude. More importantly, benefiting from the inherent self‐filtering and mechanical damping characteristics of the liquid electrolyte within the SiE microdevice, our sensor achieves an oscillation ratio of approximately 0, which means a completely oscillation‐free, clean signal output).

**TABLE 1:** Performance comparison of different high‐*g* shock sensors.

Work	Self‐powered or not	Voltage	Oscillation ratio
Yu [51]	×	≈120 mV	1.09
Lv [52]	×	≈250 mV	0.78
Shi [53]	×	≈67 mV	0.19
Choi [54]	×	≈15 mV	0.27
Li [55]	√	≈120 mV	0.62
KT1000G [56]	√	Charge signal; requires an amplifier	1.48
Our work	√	≈450 mV	0

Furthermore, we compared the signal outputs of the SiE microdevice under field live‐fire target penetration tests and laboratory Machete Hammer impacts (as detailed in Note S15). We also integrated the SiE microdevice with a System‐in‐Package chip to construct a microsystem, performing a prototype demonstration that emulates real‐world penetration layer‐counting scenarios (see Note S16). It is evident that the exceptional oscillation suppression characteristics of the SiE microdevice fundamentally overcome the severe signal adhesion issue encountered by traditional devices, demonstrating immense engineering potential for precise layer‐counting applications in hard‐target penetrating fuzes.

## 3 Conclusion

This study proposes a Sensing‐in‐Energy (SiE) microdevice featuring a microsphere‐based inertial switch embedded within the electrolyte cavity of a supercapacitor. Through an integrated structural‐functional design, the microdevice utilizes the functional interface design of the microsphere and the modulation of the shock response of the solid‐liquid coupled electrochemical system by electrolyte damping to achieve oscillation‐free sensing of high‐*g* shock while ensuring a stable energy supply for the system. The innovations and performance advancements of this work are summarized as follows:

1. A design architecture for integrated extreme shock sensing and energy supply modulated by the contact between the microsphere structure and the planar electrode within the supercapacitor electrolyte cavity is proposed. The curved contact interface between the embedded metal microsphere and the energy storage electrode achieves the concentration and enhanced modulation of contact stress, thereby amplifying the soft short‐circuit capacitive effect under high‐*g* shock and enhancing the sensing signal. Simulation and experimental tests indicate that the shock response amplitude reaches 450 mV, the measurement range exceeds 30 000 *g*, and the signal adhesion coefficient is reduced by 90.84% compared to traditional commercial accelerometers.
2. A fluid‐structure interaction (FSI)‐electrical contact‐electrochemical coupling modeling and simulation system for the microsphere‐embedded SiE microdevice is proposed. The influences of microsphere material, size, and array pattern on the sensing signal were analyzed, and a multi‐physics simulation‐driven optimization design framework based on “array dominance, material optimization, and radius regulation” was constructed, realizing the forward design of the SiE microdevice. Simulation and experimental tests show that the design error of the shock response signal amplitude does not exceed 8%, and the tunable range of the threshold covers 7000 to 24 000 *g*.
3. A micro‐nano manufacturing method for the SiE microdevice featuring high‐precision microsphere‐embedded sensing is proposed. Leveraging the precision advantage of photolithography positioning and the convenience of microsphere electroforming, high‐precision and facile manufacturing of the SiE microdevice is achieved. Repeatability tests using a Machete hammer demonstrate that the error of the shock response signal amplitude is less than 10%.

To address the critical pain points of traditional commercial accelerometers, which typically suffer from severe sensing signal oscillation under extreme impact, the proposed SiE microdevice leverages its compact solid‐liquid stacked architecture to provide superior mechanical damping. This fundamentally eliminates parasitic oscillations and endows the device with a wide measurement range and tunable thresholds, yielding remarkably pure sensing signals that successfully resolve the engineering challenge of layer‐counting failures in extreme high‐*g* environments. Looking forward, the practical deployment of this microdevice into existing weapon systems does present certain engineering challenges, notably the need for customized system‐level integration and the mitigation of electrode‐electrolyte interface aging during prolonged storage [57, 58]. Ultimately, although issues concerning long‐term durability remain to be addressed, the SiE microdevice exhibits unparalleled engineering potential for precise layer‐counting in hard‐target penetrating fuzes, driven by its exceptional signal clarity, robust shock survivability, and high level of miniaturization.

## 4 Method

### 4.1 Materials Preparation

The top and bottom electrodes were composed of ruthenium oxide. First, a mixture of ruthenium trichloride powder was dissolved in isopropanol solvent to obtain a homogeneous precursor solution after sufficient magnetic stirring. Subsequently, the precursor was applied onto a surface‐roughened titanium metal substrate using a spin‐coating process. The samples were first dried at 80°C for 10 min and then immediately placed in a high‐temperature sintering furnace and sintered at 350°C for 4 h to obtain ruthenium oxide‐based composite electrodes. The intermediate sensing layer utilized KB6165F TG150 boards, fabricated based on standard FR4‐PCB processes, with titanium microspheres welded thereon. The flange structure utilized GF212 TG130 boards, precisely formed via laser cutting technology. Interlayer connections were bonded using PUR structural adhesive, and finally, the entire microdevice was potted with epoxy resin (HASUNCAST‐3018) to ensure structural stability and insulation.

### 4.2 Microdevice Fabrication

#### 4.2.1 Stack Assembly

The fabrication of the microdevice adopted a layered stack assembly process. First, a precision dispensing system was used to apply PUR structural adhesive to the edge of the bottom electrode to bond the flange layer, followed by preliminary curing. Subsequently, PUR adhesive was applied to the flange surface, and the intermediate sensing layer was aligned and placed. To construct the electrolyte injection channel, a wedge‐shaped spacer was pre‐placed after applying PUR adhesive to the edge of the sensing layer. The top electrode was then bonded, and the assembly was left at room temperature until the PUR adhesive was fully cured. Upon completion of curing, the spacer was removed to form the liquid injection hole.

#### 4.2.2 Liquid Injection and Packaging

The assembled microdevice was immersed in a container containing 50 wt.% sulfuric acid electrolyte and placed within a vacuum injection machine for a two‐stage vacuum impregnation process. The specific procedure was as follows: first, a vacuum was drawn and held for 5 min to exhaust air from the pores; subsequently, the system was restored to a half‐pressure state, and a vacuum was drawn and held again for 5 min, utilizing the pressure difference to force the electrolyte to fully fill the internal microstructures of the microdevice. After liquid injection, the microdevice underwent shell assembly and was sealed with epoxy resin. Finally, it was cured at a constant temperature of 55°C for 1 h to complete the preparation of the SiE microdevice.

### 4.3 Experimental Testing

This study employed a calibrated Machete hammer shock test system to evaluate the dynamic response characteristics of the SiE microdevice. The testing covered three typical high‐*g* energy levels: 10 000, 20 000, and 30 000 *g*. In the experiment, the SiE microdevice under test was fixed onto the horizontal test surface at the tip of the hammer and connected to a digital oscilloscope and a DC regulated power supply via shielded cables, respectively, to construct a complete signal acquisition loop. Before the experiment, the Machete hammer was lifted to the ratchet position corresponding to the target *g*‐value and locked, endowing the system with specific initial gravitational potential energy. Subsequently, the oscilloscope was set to single‐trigger mode to capture transient signals. Prior to the formal impact, the DC power supply was disconnected, and the Machete hammer was released. Driven by the counterweight, the hammer head fell freely and impacted the base, instantaneously generating a shock load of up to tens of thousands of times the gravitational acceleration. At this moment, the oscilloscope synchronously recorded and saved the transient voltage response signal of the microdevice to complete the test.

## Author Contributions

Z.Z., Y.W., and K.D. conceived the idea and designed the project. Z.Y., X.W., and K.D. supervised the project. Z.Z. and Y.W. constructed the theoretical framework and designed the experiments. Z.Z., K.L., and Y.Y. completed the finite element simulations, Z.Z. and S.L. conducted the experimental tests. Z.Z, Y.W., and K.D. prepared the figures and wrote the manuscript. All the authors commented on the manuscript.

## Conflicts of Interest

The authors declare no conflicts of interest.

## Supporting information




**Supporting File**: advs75530‐sup‐0001‐SuppMat.docx.

## Data Availability

The data that support the findings of this study are available from the corresponding author upon reasonable request.
