# Supplementary material for: Microsphere‐Modulated Sensing‐in‐Energy Supercapacitor With Self‐Filtering Ultra‐Large Signal Under High‐g Shocks
Source: Adv Sci (Weinh). 2026 May 6;13(42):e75530. doi: 10.1002/advs.75530 (PMC13335590; doi:10.1002/advs.75530)
Supplement: Supplementary file 1 — Supporting File: advs75530‐sup‐0001‐SuppMat.docx. [file ADVS-13-e75530-s001.docx]

**Supplementary Material**

**Microsphere-Modulated Sensing-in-Energy Supercapacitor with Self-filtering Ultra-large Signal under High-*g* Shocks**

Zhihao Zheng^1,†^, Yiqun Wang^1,†^, Kaiyou Liu^1^, Shancheng Luan^1^, Yajiang Yin^1^, Xiaofeng Wang^1, 🖂^, Keren Dai^2, 🖂^ and Zheng You^1^

*1 Department of Precision Instrument, Tsinghua University, Beijing 100084, China*

*2 School of Mechanical Engineering, Nanjing University of Science and Technology, Nanjing 210094, China*

*† These authors contributed equally to this work.*

**^🖂^** *The Corresponding authors: Xiaofeng Wang (*[xfw@mail.tsinghua.edu.cn](mailto:xfw@mail.tsinghua.edu.cn)*), Keren Dai (*[dkr@njust.edu.cn](mailto:dkr@njust.edu.cn)*)*

**Supplementary Note 1: Energy storage characteristics of the SiE microdevice**

**Supplementary Note 2: The relationship between electrolyte viscosity and microsphere dynamics**

**Supplementary Note 3: Assumptions of the Multi-physics Model**

**Supplementary Note 4: Comparison of macroscopic contact areas between spherical and planar contacts**

**Supplementary Note 5: Composition of contact current**

**Supplementary Note 6: Dynamic model of equivalent circuit for supercapacitor**

**Supplementary Note 7: Force analysis of a microsphere moving in a fluid**

**Supplementary Note 8: Analysis of fluid damping on a moving microsphere**

**Supplementary Note 9: Complex relationship between response signal amplitude and gap size at small gaps**

**Supplementary Note 10: Comparison between the phase of fluid-structure interaction and the inertial force phase under small-gap conditions**

**Supplementary Note 11: Structure and working principle of the Machete hammer**

**Supplementary Note 12: Comparison between experimental results and simulation curves of the response signal**

**Supplementary Note 13: Effect of incomplete electrolyte filling on the amplitude of shock response signals**

**Supplementary Note 14: Definition of the layer coefficient as a characterization of the degree of signal adhesion**

**Supplementary Note 15: Signal comparison of the SiE microdevice under live-fire penetration and laboratory Machete Hammer impacts**

**Supplementary Note 16: Prototype demonstration of the SiE microdevice for penetration layer-counting scenarios**

**Supplementary Note 1: Energy storage characteristics of the SiE microdevice**

The SiE modules with different voltage ratings all exhibit excellent electrochemical energy storage characteristics. Fig. S1(a) presents the galvanostatic charge-discharge (GCD) curves of the SiE modules with different voltages at 20 mA. Fig. S1(b) displays the GCD characteristics of the 5V module at currents of 5 mA, 10 mA, and 20 mA, while Fig. S1(c) illustrates the cyclic charge-discharge performance of the 5V module. The Equivalent Series Resistance (ESR) and capacitance of the different modules are shown in Fig. S1(d). It is evident that the SiE microdevice features low ESR (<1.5 Ω) and high capacitance (>3 mF), providing a solid guarantee for the stable energy supply of the system.

**
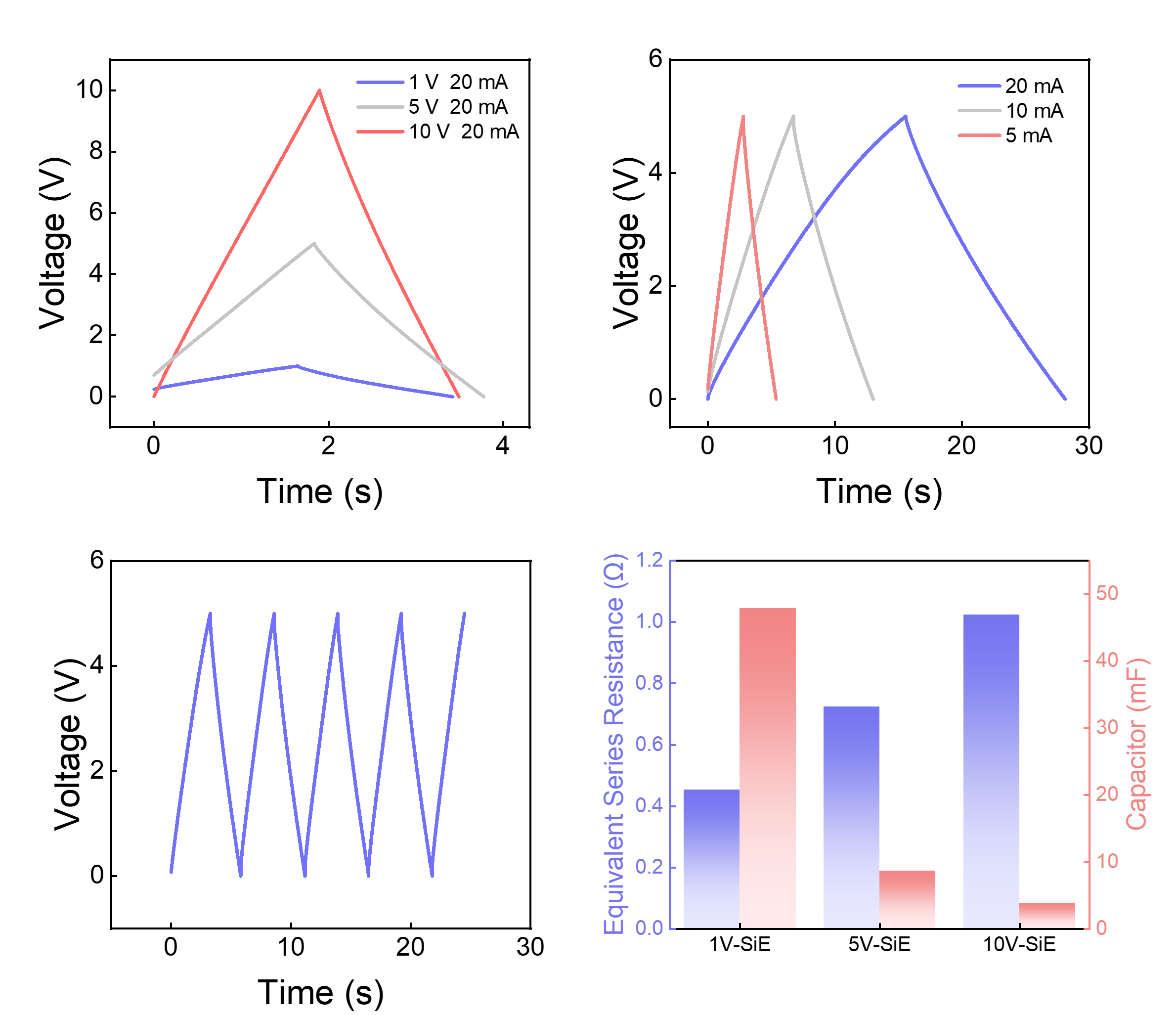
**

**Figure S1** (a) Single-cycle galvanostatic charge-discharge (GCD) curves of the 1 V, 5 V, and 10 V voltage modules at 20 mA. (b) GCD curves of the 5 V voltage module at currents of 5 mA, 10 mA, and 20 mA. (c) Cyclic GCD curves of the 5 V voltage module at 20 mA. (d) Comparison of the ESR and capacitance of the 1 V, 5 V, and 10 V voltage SiE-modules.

**Supplementary Note 2: The relationship between electrolyte viscosity and microsphere dynamics**

From a structural dynamics perspective, the 'four-beam + microsphere' assembly can be simplified as a classical single-degree-of-freedom second-order damped oscillator. Its governing equation of motion is given by:

$$m\frac{d^{2}x}{dt^{2}}+c\frac{dx}{dt}+kx=F_{ext}(t)$$

where $m$ is the equivalent mass of the microsphere and the beams, $k$ is the equivalent stiffness of the four-beam structure, $F_{ext}(t)$ is the transient inertial force induced by the high-*g* impact, and $c$ is the equivalent viscous damping coefficient provided by the electrolyte. The fluid viscosity $\mu$ directly determines the magnitude of $c$. We introduce a critical dimensionless parameter, the damping ratio $\zeta$:

$$\zeta=\frac{c}{2\sqrt{mk}}$$

Variations in the electrolyte viscosity directly govern the oscillatory state of the system:

- When the internal cavity is filled with air, the fluid viscosity $\mu$ is negligible, resulting in a small damping coefficient $c$ and a damping ratio $\zeta\ll1$ (underdamped). The microsphere would repeatedly strike the bottom electrode, generating multiple 'drop-and-recovery' signals, which would cause severe logical misjudgments during layer-counting recognition.
- When the electrolyte viscosity is optimal, $\zeta\approx1$, and the system operates in a critically damped state. After triggering the short-circuit, the microsphere returns to its initial equilibrium position without oscillation. Even if slight residual movement occurs upon return, it cannot reach the bottom electrode to trigger another short-circuit, ensuring the device outputs an exceptionally clean single pulse.
- When the electrolyte viscosity is excessively high ($\zeta>1$, overdamped), the substantial damping dissipates the microsphere's kinetic energy before it can reach the bottom electrode, failing to trigger the contact short-circuit and generate a signal.

we have further supplemented simulations regarding the effect of electrolyte viscosity, as illustrated in Figure S2. As can be intuitively observed, with the increase in electrolyte viscosity, the mechanical oscillations of the microsphere and the four-arm beam are effectively suppressed. Furthermore, when the electrolyte viscosity is sufficiently high, it can even affect the occurrence of the contact short-circuit. The above describes the effect of electrolyte viscosity on the dynamic characteristics of the microsphere.


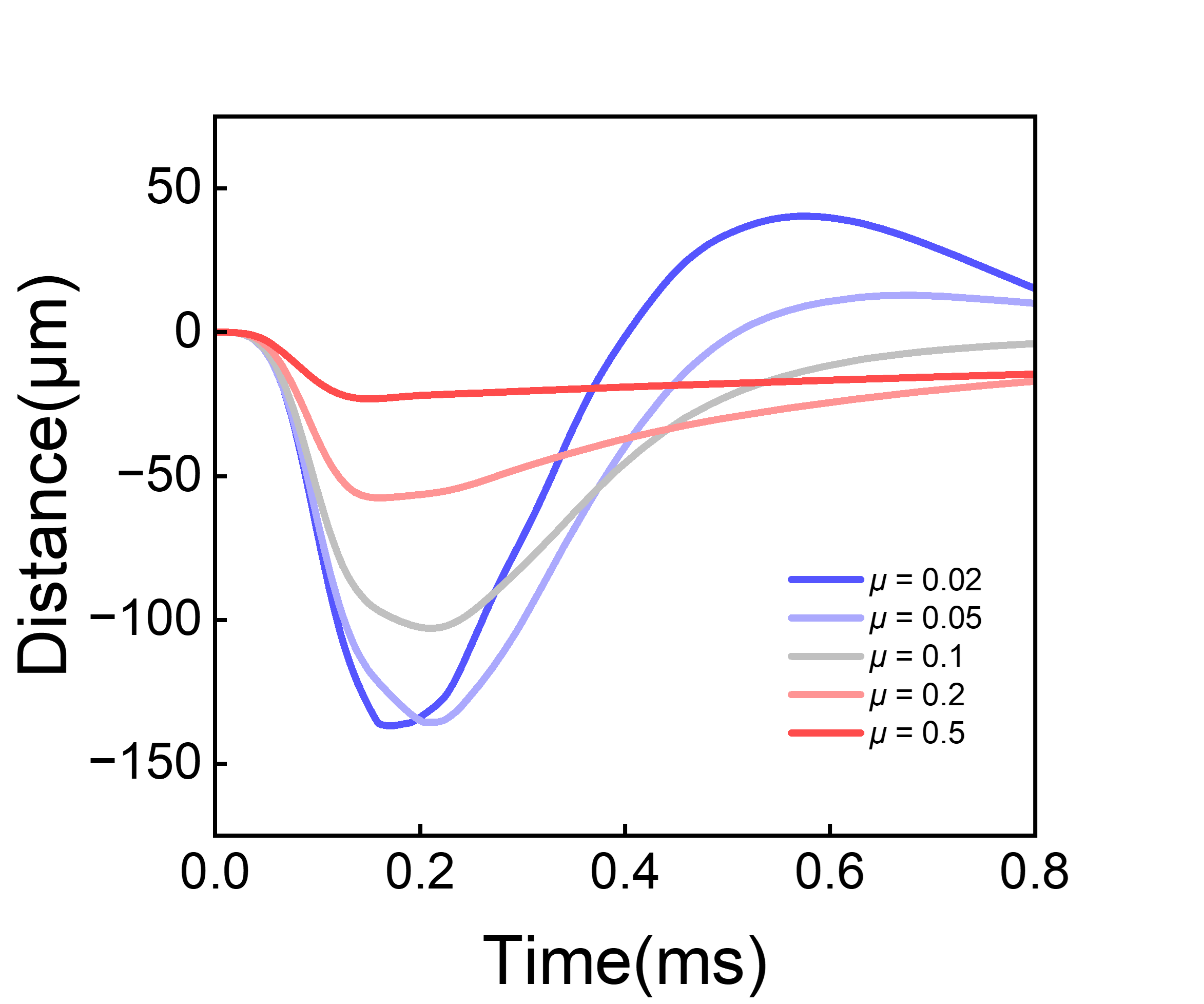


**Figure S2**. Relationship between electrolyte viscosity and microsphere displacement

**Supplementary Note 3: Assumptions of the Multi-physics Model**

1. Equivalent loading hypothesis of high-*g* shock

During an actual high-*g* impact, materials inevitably undergo an extremely brief period of microscopic deformation accumulation. Consequently, the acceleration rises smoothly from zero, taking the form of a Gaussian function [1-4]. In our multi-physics simulation, the high-*g* impact load is modeled as the product of a Gaussian function and a smoothed step function. Specifically, the Gaussian function is utilized to represent the realistic acceleration profile of a high-speed impact, while the smoothed step function is introduced to eliminate the non-physical, non-zero acceleration artifact of the Gaussian curve at initial moment. This approach not only strictly adheres to real physical laws but also effectively suppresses high-frequency numerical oscillations during the solving process, thereby accurately reproducing the transient loading conditions under extreme high-*g* impacts.

1. Laminar flow assumption for the electrolyte

The fluid regime is fundamentally governed by the transient Reynolds number, and the assumption of laminar flow is dictated by the internally confined space of the SiE microdevice and the physical properties of the electrolyte [5-7]. In our SiE microdevice, the characteristic length, which corresponds to the dynamically narrowing gap between the moving microsphere and the cavity boundary, is strictly on the micrometer scale. Although the high-*g* impact induces a relative velocity of the internal microsphere, the maximum velocity is strictly bounded by the fluid's viscous damping and the exceptionally short travel distance prior to contact closure. Consequently, the transient Reynolds number （The calculated result is approximately 460） remains far below the critical threshold for turbulence (>1000). Therefore, adopting the laminar flow assumption is physically imperative.

1. Assumptions of electrical contact at the interface

Regardless of the precision of the fabrication processes for the microsphere and electrode surfaces, they inevitably consist of countless rough asperities at the microscopic scale [8]. When modeling the contact interface between the microsphere and the electrode, explicitly resolving the rough geometry of the contacting surfaces would require an excessively high computational mesh density. Furthermore, strongly coupling such a dense mesh with the highly nonlinear transient fluid-structure interaction solver would induce severe convergence issues. Therefore, we explicitly assume in our model that the macroscopic contact surfaces are ideally flat, while the actual microscopic surface roughness is accounted for electrically via the Cooper-Mikic-Yovanovich (CMY) correlation. The CMY correlation accurately bridges the gap between macroscopic mechanics and microscopic electrodynamics [9]. It assumes that under high-*g* transient impact, the microscopic asperities will undergo plastic deformation [10]. In our SiE microdevice, the local transient contact stress generated at the moment of microsphere impact is immensely high, far exceeding the yield strength of the constituent metals and their oxide materials. Consequently, the asperities at the collision interface inevitably undergo plastic yielding within an extremely short timeframe, which aligns perfectly with the fundamental assumption of the CMY theory. Furthermore, by implementing the CMY model, the contact conductance is dynamically coupled with the transient contact pressure extracted from the FSI module. This allows our model to accurately predict the sharp drop in contact resistance as the actual contact area increases under high-*g* impact, thereby ensuring both the physical fidelity of the electrical signals and the numerical convergence of the multi-physics model.

**Supplementary Note 4: Comparison of macroscopic contact areas between spherical and planar contacts.**

As shown in Fig. S3, multi-physics simulation indicates that under identical contact gap and high-*g* shock conditions, the planar structural contact exhibits a larger macroscopic contact area compared to the microsphere structural contact.


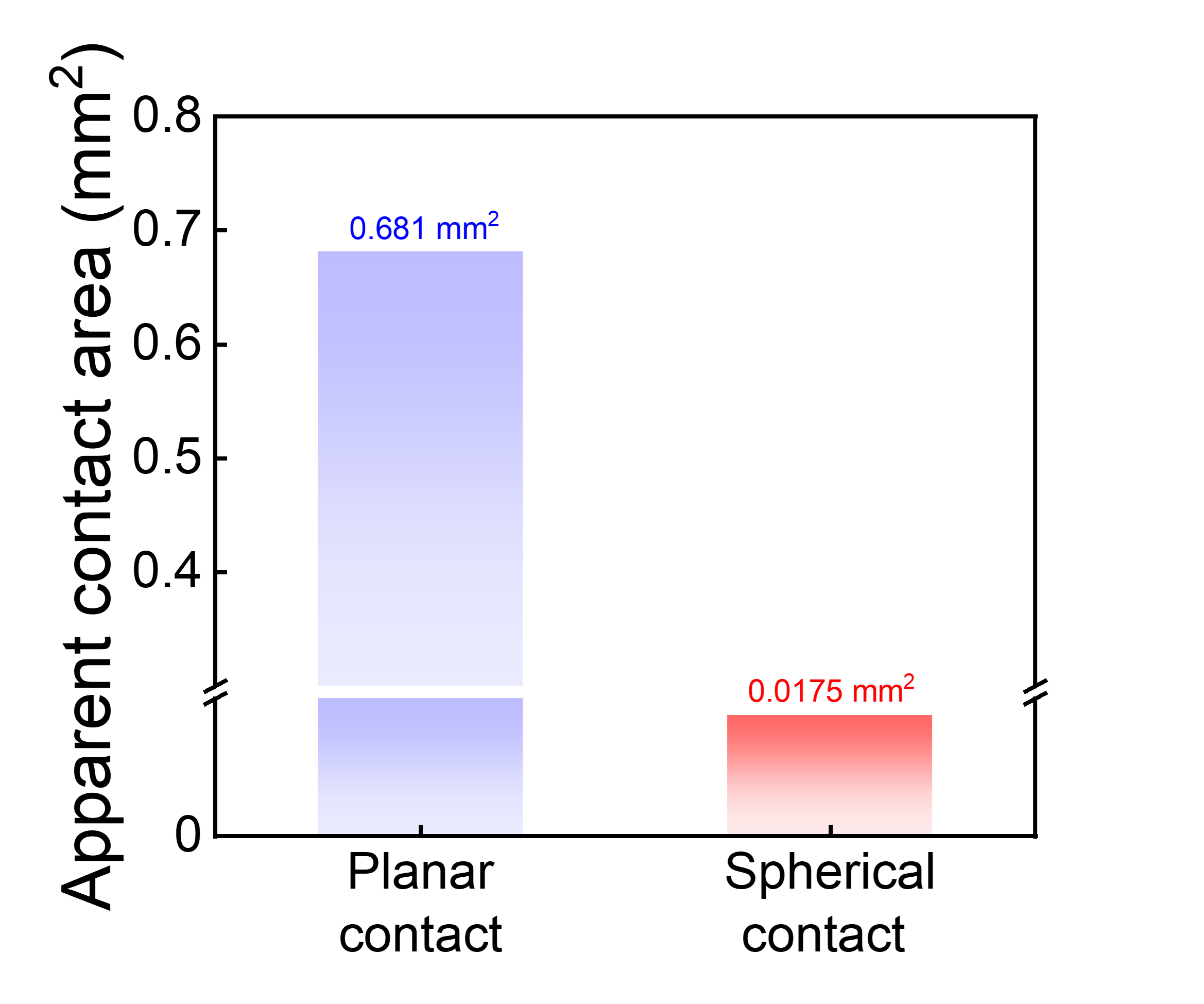


**Figure S3** Comparison of the macroscopic contact areas between the planar structure contact and the microsphere structure contact

**Supplementary Note 5: Composition of contact current**

As shown in Fig. S4, the contact current is composed of conduction current and displacement current. However, the proportion of displacement current is negligible; thus, it is ignored in subsequent contact current calculations, where only the influence of conduction current is considered.


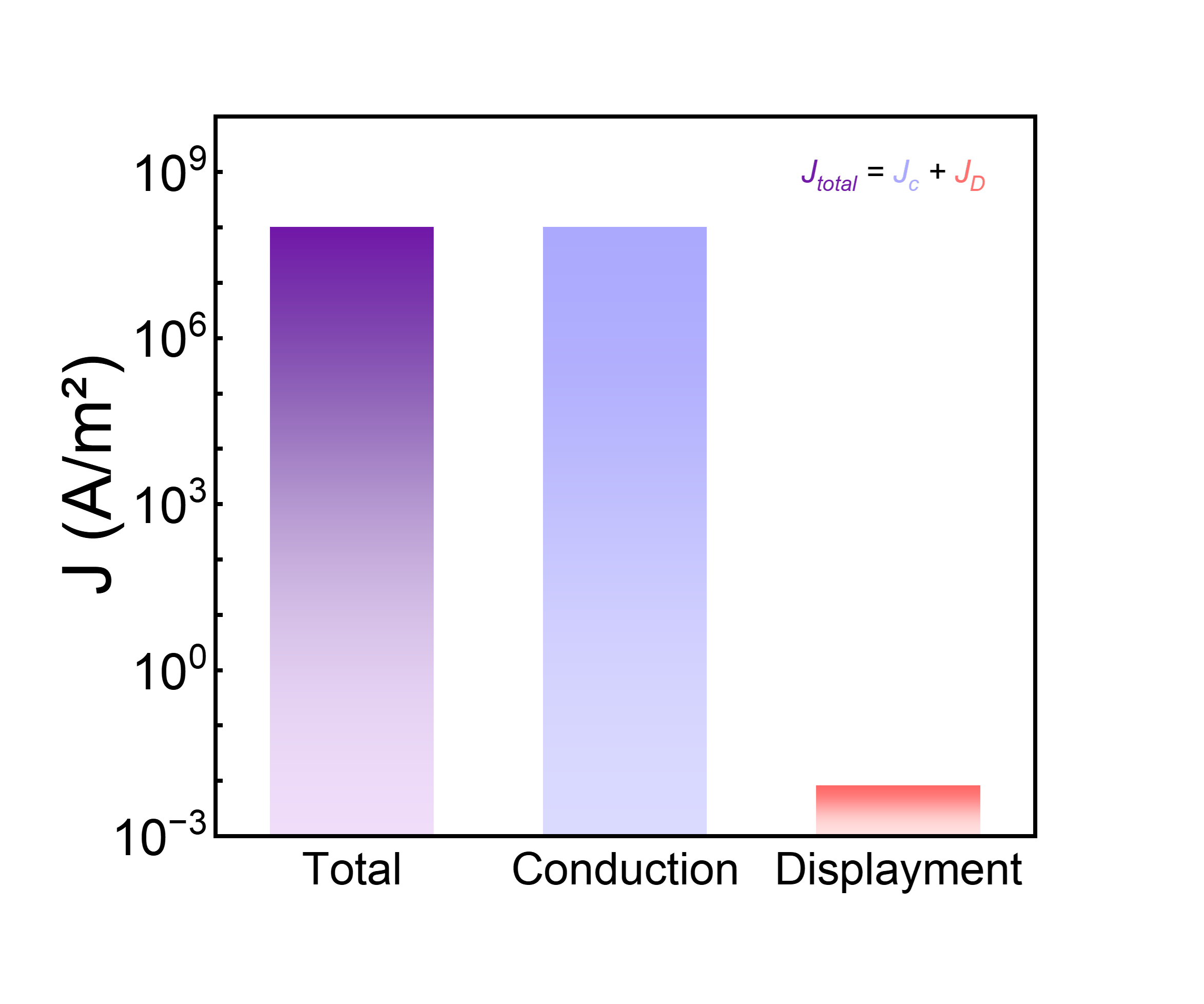


**Figure S4** Contact current is composed of conduction current and displacement current.

**Supplementary Note 6: Dynamic model of equivalent circuit for supercapacitor**

As shown in Fig. S5, based on the simplified series RC model of the supercapacitor, a polarization branch (*R*_p_-*C*_p_) is introduced. Furthermore, to incorporate the force-sensing mechanism into the circuit model, the original fixed internal resistance is replaced by a variable internal resistance *R*_i_, and a variable leakage resistance *R*_s_ is added in parallel externally to simulate the "transient short-circuit" behavior.


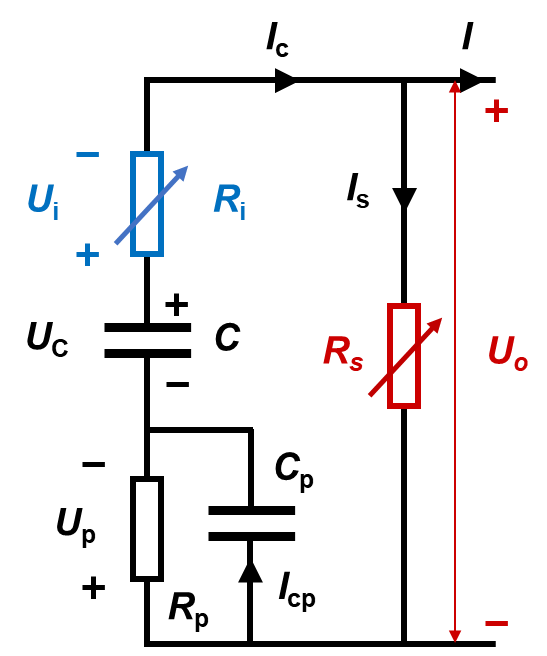


**Figure S5** Dynamic model of equivalent circuit for supercapacitor

**Supplementary Note 7: Force analysis of a microsphere moving in a fluid**

As shown in Fig. S6, under high-*g* shock, in the reference frame of the bottom electrode, the forces acting on the microsphere moving in the electrolyte primarily include the inertial force, gravity, electrolyte drag force, buoyancy, and the elastic restoring force of the beam.


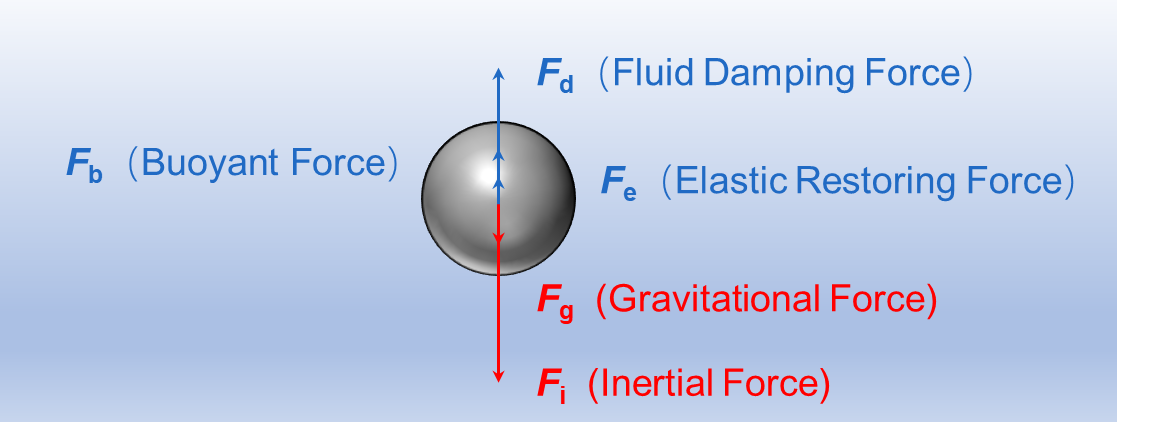


**Figure S6** Schematic of forces acting on the microsphere moving in the electrolyte under high-*g* shock.

**Supplementary Note 8: Analysis of fluid damping on a moving microsphere**

(1) Fluid damping force acting on the microsphere when moving far from the contact surface:

$$\text{F}_{\text{drag}}\text{ }\text{=}\text{ }\text{6π}\text{μRv}$$

Where $\text{F}_{\text{drag}}$represents the fluid viscous damping force, 𝜇 represents the fluid dynamic viscosity, 𝑅 represents the microsphere radius, and 𝑣 represents the instantaneous velocity of the microsphere relative to the fluid.

(2) When the microsphere approaches the bottom electrode, the fluid underneath cannot be drained instantaneously, generating a significant squeeze-film damping effect. In this regime, the damping force is primarily described by Reynolds lubrication theory:

$$\text{F}_{\text{squeeze}}\text{ }\text{≈}\text{ }\frac{\text{βπ}\text{μ}\text{R}^{\text{2}}\text{v}}{\text{h}}$$

Where $\text{F}_{\text{squeeze}}$ represents the squeeze-film damping force, 𝜇 represents the fluid dynamic viscosity, 𝑅 represents the microsphere radius, 𝑣 represents the instantaneous normal approach velocity of the microsphere toward the wall, ℎ represents the instantaneous vertical gap between the bottom apex of the microsphere and the electrode surface, and 𝛽 is a geometric correction factor depending on specific boundary condition assumptions.

**Supplementary Note 9: Complex relationship between response signal amplitude and gap size at small gaps**

As shown in Fig. S7, when the microsphere radius is large and the initial contact gap is small, the amplitude of the response signal exhibits a "high-low-high" variation as the contact gap increases.


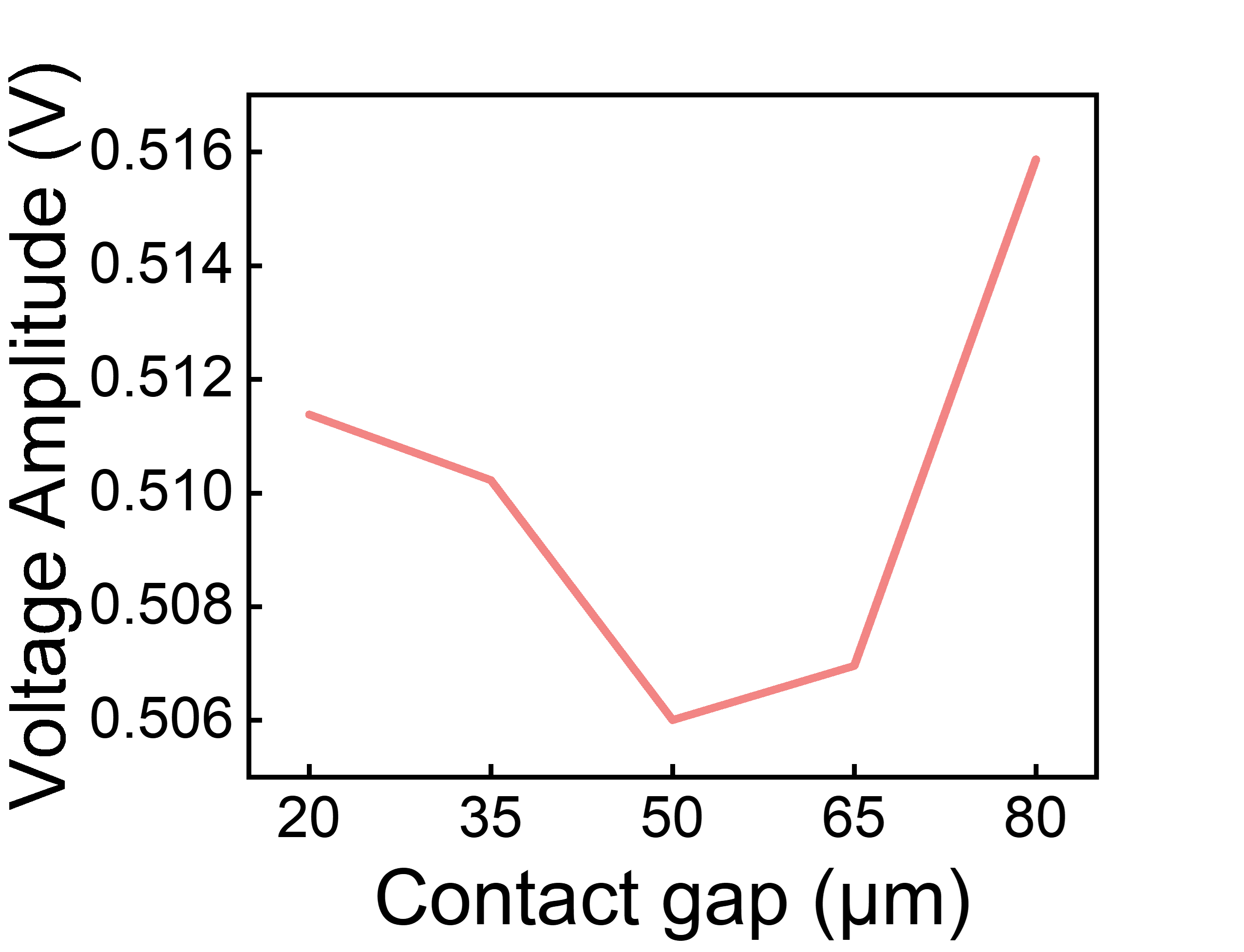


**Figure S7** Complex variations of the response voltage amplitude under small contact gap conditions

**Supplementary Note 10: Comparison between the phase of fluid-structure interaction and the inertial force phase under small-gap conditions**

As shown in Fig. S8, when the microsphere radius is large and the total gap height is small (i.e. a small contact gap), as the total gap height increases, the moment of maximum displacement gradually moves away from the moment of peak acceleration, and the dominant role of inertial force in the contact pressure gradually weakens.


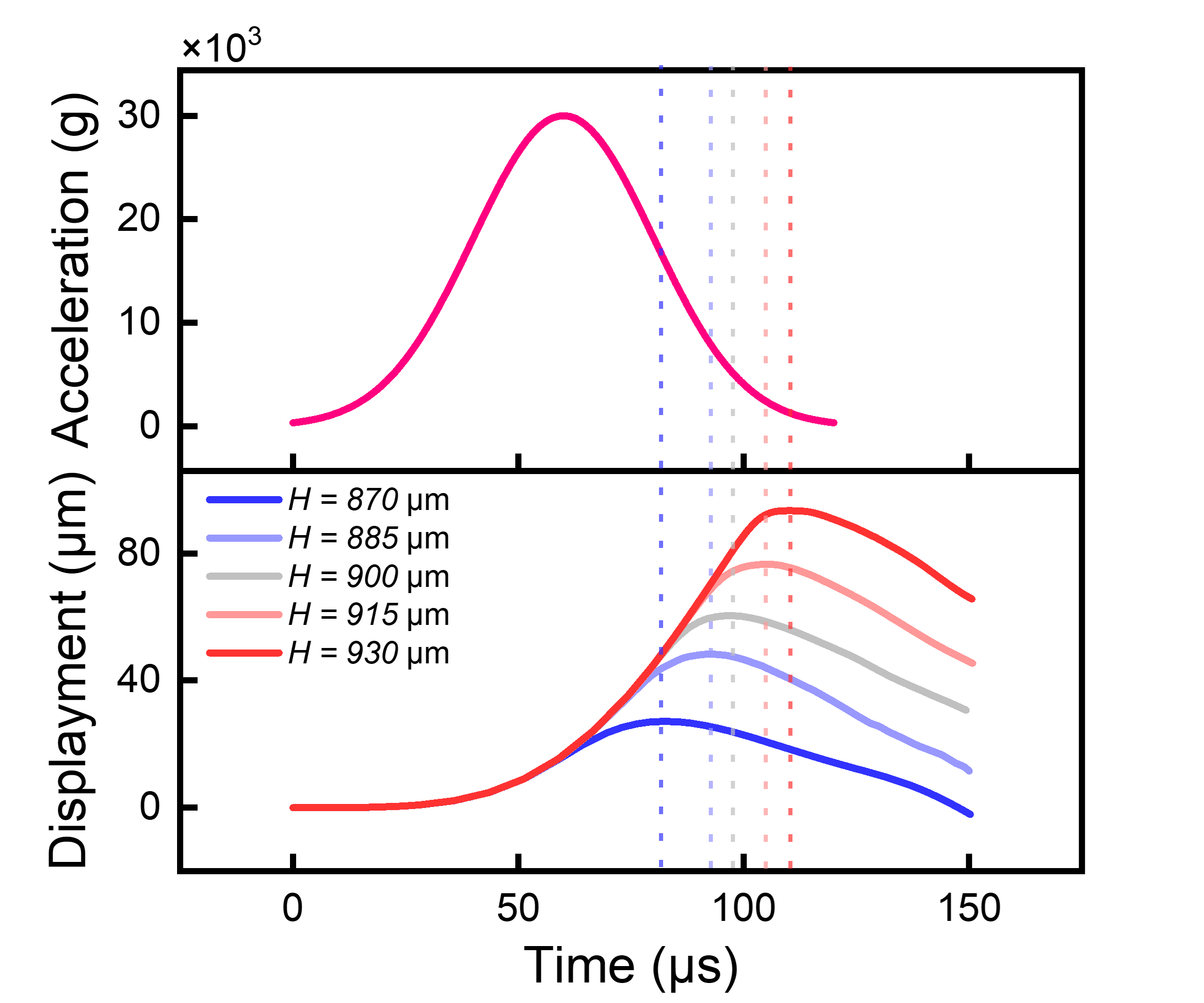


**Figure S8** Phase comparison between the contact moment and the peak inertial acceleration moment with a large microsphere radius and small total gap height (small contact gap).

**Supplementary Note 11: Structure and working principle of the Machete hammer**

The Machete hammer is a mechanical shock testing device capable of generating transient high-*g* shock loads. It simulates high-*g* environments during collisions, with peak accelerations reaching tens of thousands of *g*, making it a standard laboratory apparatus for simulating high-*g* shock environments (Fig. S9(a)). Fig. S9(b) illustrates its working principle. The hammer is lifted to a ratchet position corresponding to the target *g*-value and locked to endow the system with specific initial gravitational potential energy. Upon release, driven by counterweights, the hammer head falls freely and impacts the base, instantaneously generating a shock load of up to tens of thousands of times the gravitational acceleration.


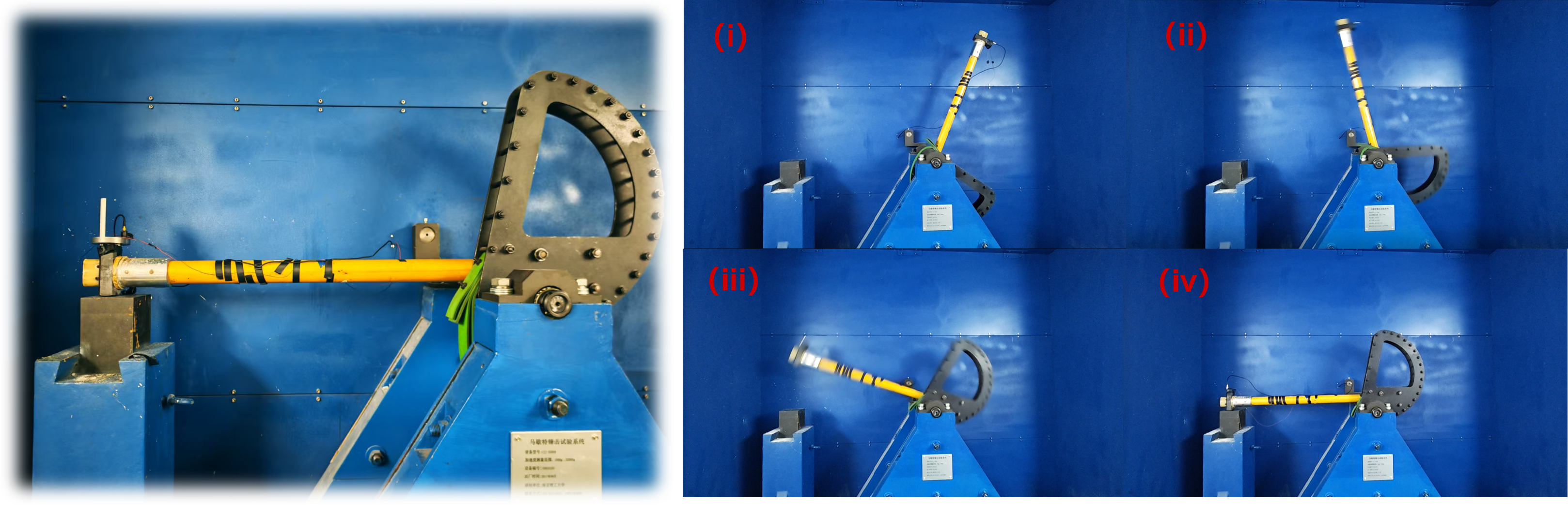


**Figure S9** Structure and working principle of the Machete hammer (a) Photograph of the Machete hammer; (b) Working principle of the Machete hammer

**Supplementary Note 12: Comparison between experimental results and simulation curves of the response signal**

The experimental test curves and multi-physics simulation results are shown in Fig. S10. Under a high-*g* shock environment of 30,000 *g*, the amplitude and pulse width of both signals are essentially identical, demonstrating good consistency between the simulation and experiment.


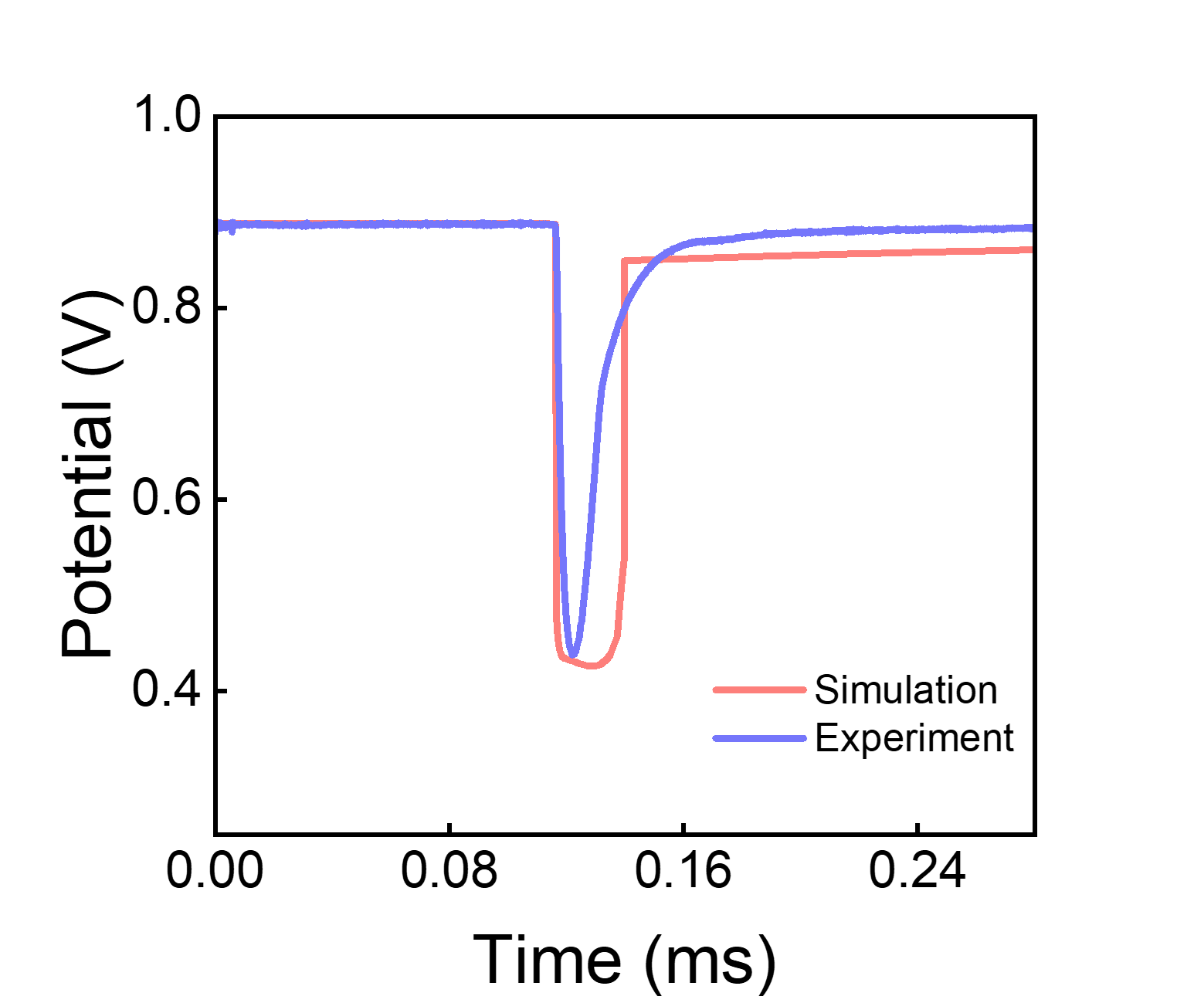


**Figure S10** Comparison of the measured response voltage curve of the SiE microdevice under 30,000 *g* shock with the simulation curve using identical structural parameters.

**Supplementary Note 13: Effect of incomplete electrolyte filling on the amplitude of shock response signals**

As shown in Fig. S11, when the electrolyte cavity of the SiE microdevice is not 100% filled with electrolyte, the amplitude of the response signal under high-*g* shock increases significantly compared to the 100% filled condition.


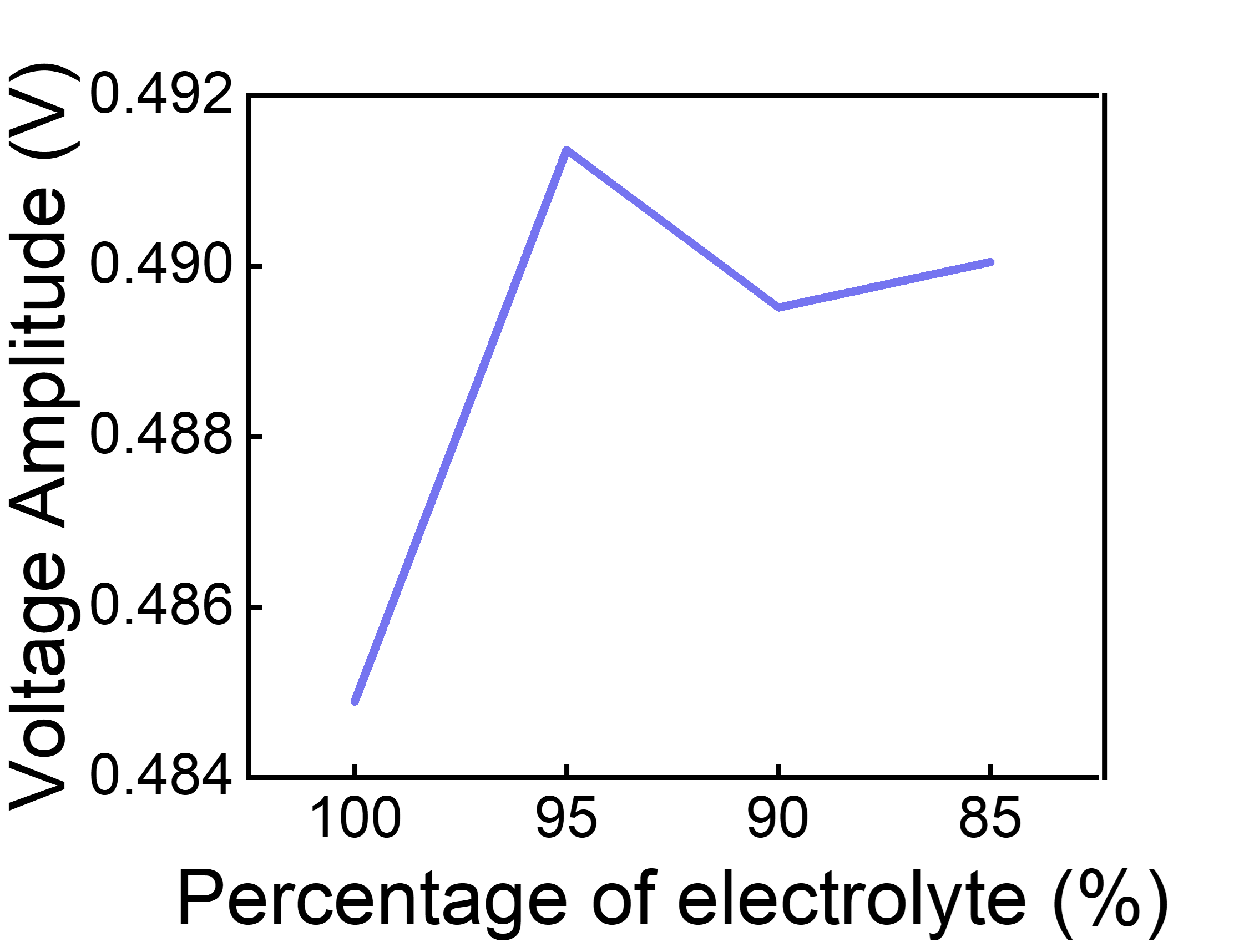


**Figure S11** The response signal amplitude is larger under incomplete electrolyte filling conditions compared to complete filling.

**Supplementary Note 14: Definition of the layer coefficient as a characterization of the degree of signal adhesion**

The layer coefficient is adopted to evaluate the degree of adhesion of the shock response signal. This coefficient reflects the oscillation extent of the signal, where a larger value indicates more severe signal jitter.

$$\text{V}_{\text{i}}\text{ }\text{=}\text{ }\frac{\sum_{\text{k}\text{=1}}^{\text{n}} \text{|}\text{V}_{\text{k}\text{+1}}\text{-}\text{V}_{\text{k}}\text{|}}{\text{N}_{\text{i}}\text{T}_{\text{i}}}$$

Where $V_{i}$represents the normalized response voltage value at the i-th sampling point, $N_{i}$represents the total number of sampling points, and $T_{i}$represents the sampling frequency.

**Supplementary Note 15: Signal comparison of the SiE microdevice under live-fire penetration and laboratory Machete Hammer impacts**

The SiE microdevice proposed in our research does exhibit slight differences under actual operating conditions (i.e., live-fire target penetration) compared to the laboratory test results. The signal comparison is illustrated in Figure S12.


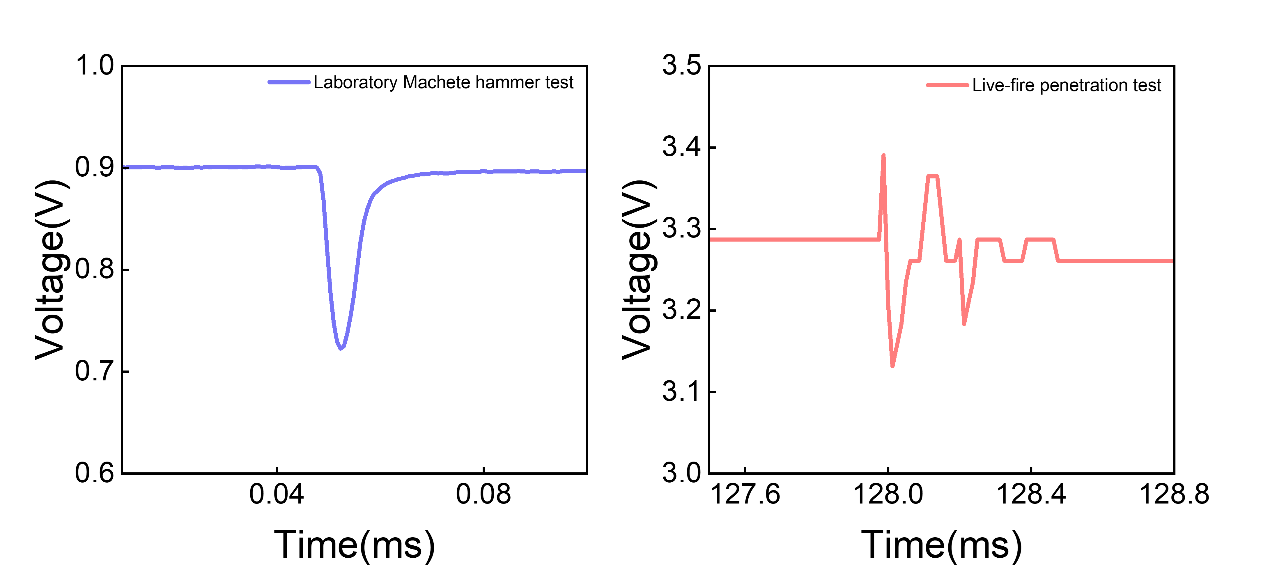


**Figure S12** Signal comparison of the SiE microdevice. (a) Laboratory Machete hammer shock test; (b) Field live-fire target penetration test.

First, upward pulses emerge in the signal of the SiE microdevice at the exact moment of live-fire penetration. This occurs because the shock experienced by the projectile during actual penetration is not a perfectly ideal one-dimensional axial load. Due to slight deflections in the projectile's attitude, strong three-dimensional transient stresses are generated upon impact. Under this complex, multi-axial extreme inertial force field, ions within the electrolyte experience severe multi-directional surges and violently impact the microstructures of the porous electrodes. This transient lateral or transverse ion flushing instantaneously disrupts the local charge equilibrium of the electric double layer, resulting in short-term upward voltage pulses in the device's output during the shock [11,12]. Furthermore, under the extreme high-overload conditions of penetration, mechanical chatter among the internal components of the fuze system can induce microsecond-level abrupt changes in the dynamic contact resistance at connection interfaces. Simultaneously, transient electromagnetic crosstalk generated by surrounding electronic components under shock may couple into the measurement circuit of the SiE microdevice, which also contributes to these slight voltage fluctuations.

Second, unlike the single signal peak generated by a single strike of the Machete hammer in the laboratory, the SiE microdevice exhibited two distinct signal peaks during a single live-fire penetration event. This is primarily attributed to the non-uniform structural and mechanical response of the finite-thickness double-reinforced concrete target. The first peak occurs during the initial "cratering phase" of penetration, when the high-speed projectile forcefully squeezes into and violently crushes the front-surface concrete while rupturing the front-layer steel reinforcement mesh, generating massive initial penetration resistance. The second peak emerges as the projectile approaches the rear surface of the target. Due to the loss of frontal material confinement, the target's failure mode transitions from internal tunneling to rear integral plugging. The projectile must overcome the target's ultimate tensile and shear strengths, thereby generating the second signal peak [2,13,14].

**Supplementary Note 16: Prototype demonstration of the SiE microdevice for penetration layer-counting scenarios**

we have not only considered but also preliminarily accomplished system-level integration and conducted a proof-of-concept demonstration that reflects a real-world penetration environment. Specifically, we integrated the SiE microdevice proposed in this study with a System-in-Package (SiP) chip to construct a functional microsystem prototype. The circuit schematic framework of the SiE microsystem is shown in Figure S13.


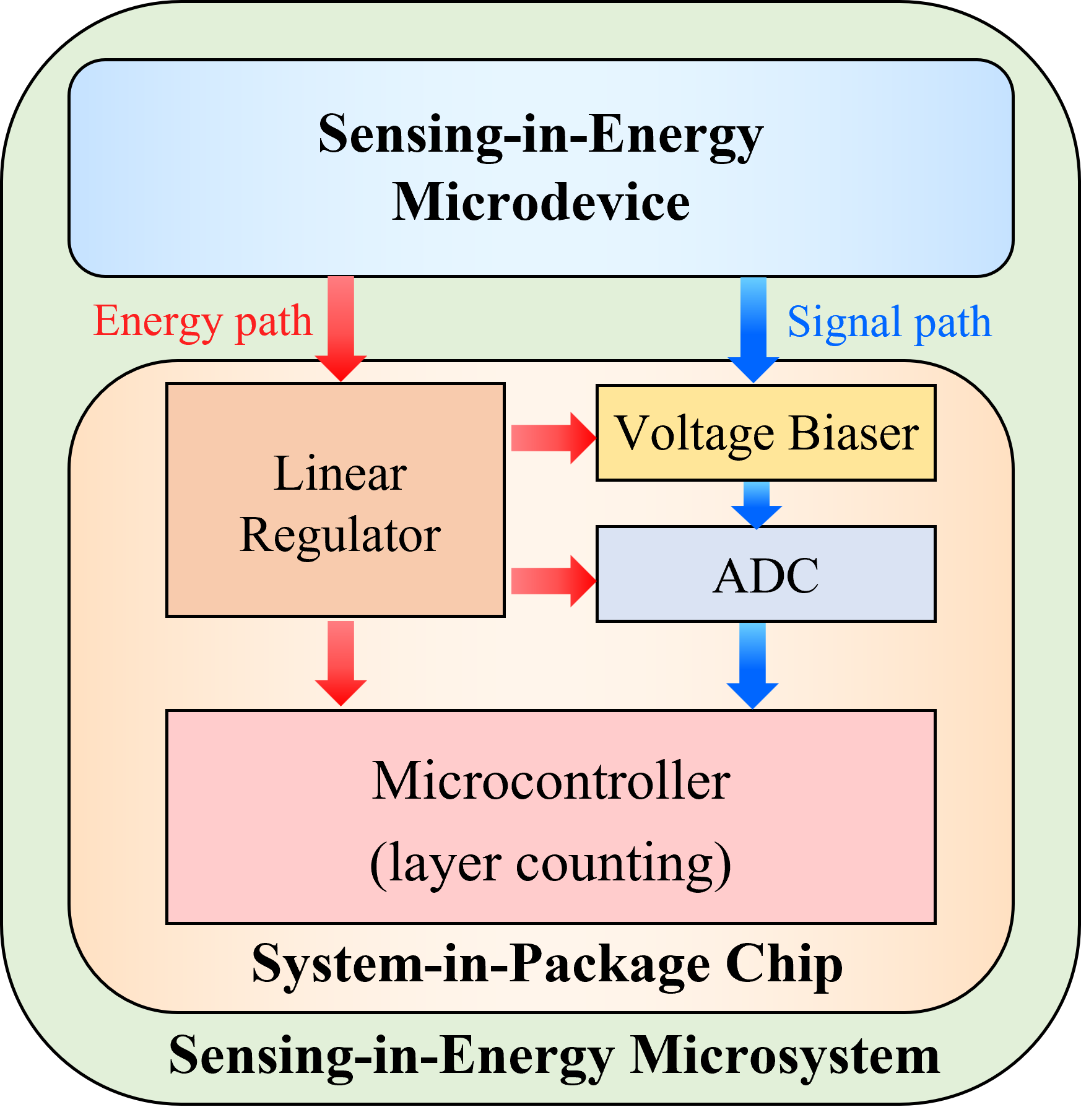


**Figure S13**. Circuit schematic framework of the SiE microsystem.

This system is capable of acutely sensing extreme high-*g* shocks and successfully executing layer-counting and detonation decision-making during the penetration process. The actual layer-counting performance obtained from the prototype demonstration is illustrated in Figure S14.


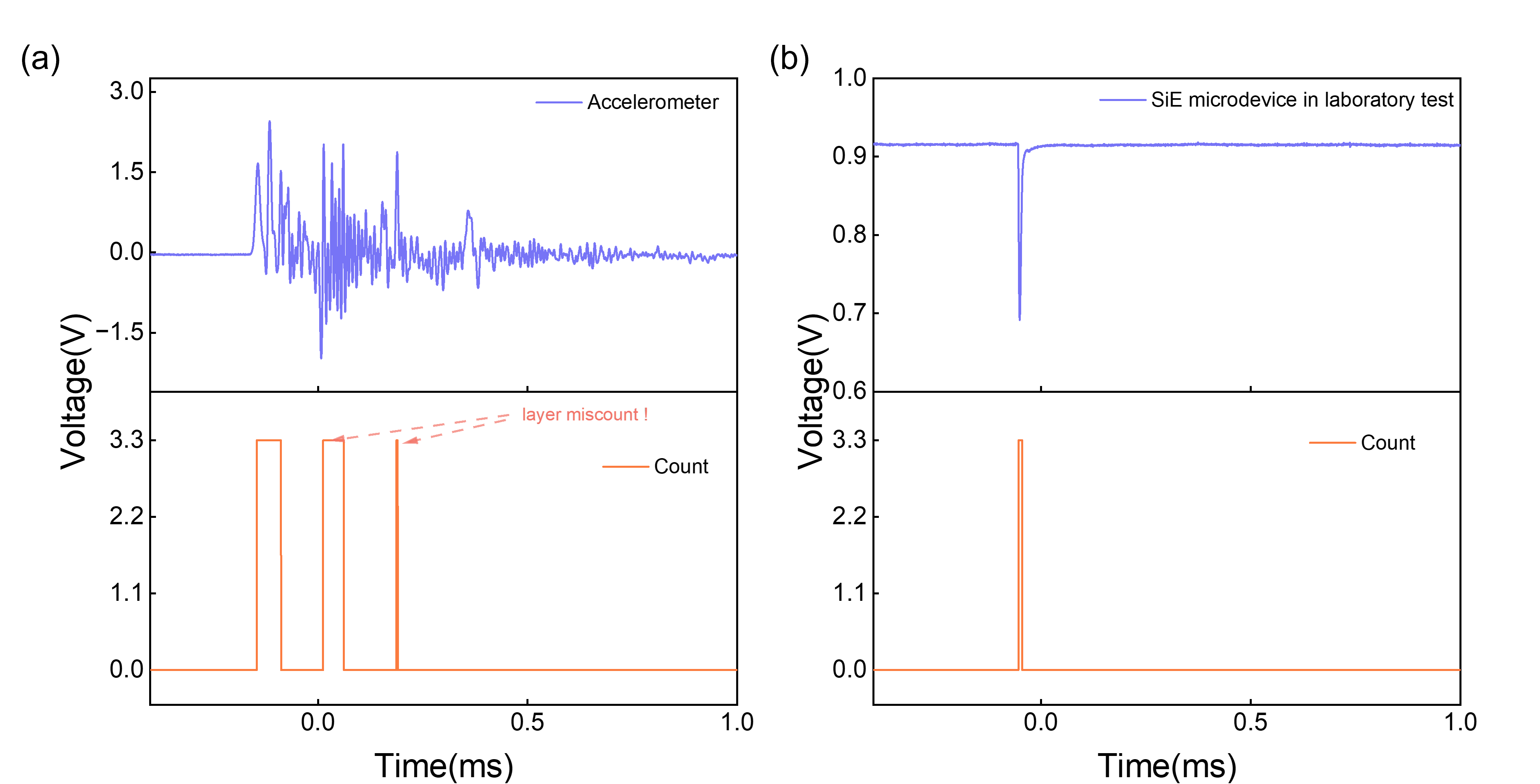


**Figure S****14** Layer-counting demonstration of the commercial accelerometer and the SiE microdevice. (a) commercial accelerometer; (b) our SiE microdevice.

First, in the laboratory Machete hammer impact test, the microsystem integrated with the SiE microdevice exhibited exceptional noise immunity. Its output signal remained smooth without obvious oscillations, thereby ensuring the precise execution of the layer-counting algorithm. In stark contrast, the commercial reference accelerometer suffered from severe vibrational fluctuations under the same shock conditions, resulting in a signal contaminated with numerous high-frequency pulse peaks. This severe distortion can easily induce "false layer-counting" errors in the algorithm, which would critically interfere with the final detonation decision of the fuze system.

Furthermore, to fully validate the system's reliability under actual operating conditions, we comparatively evaluated the layer-counting performance of the microsystem between the laboratory Machete hammer test and the live-fire target penetration test (as shown in Figure S15). To address the unique "dual-peak" physical feature inherent to live-fire penetration through reinforced concrete targets, we adaptively optimized our layer-counting strategy. The results demonstrate that, even in the extremely harsh environment of live-fire penetration, the sensing-in-energy integrated microsystem driven by the SiE microdevice can successfully overcome the interference of high-frequency mechanical noise and complex multi-axial stresses to achieve high-fidelity, accurate layer-counting for actual live-fire signals. This proof-of-concept demonstration thoroughly validates the immense application potential and engineering value of the SiE microdevice in real-world weapon microsystems such as hard-target penetration fuzes.


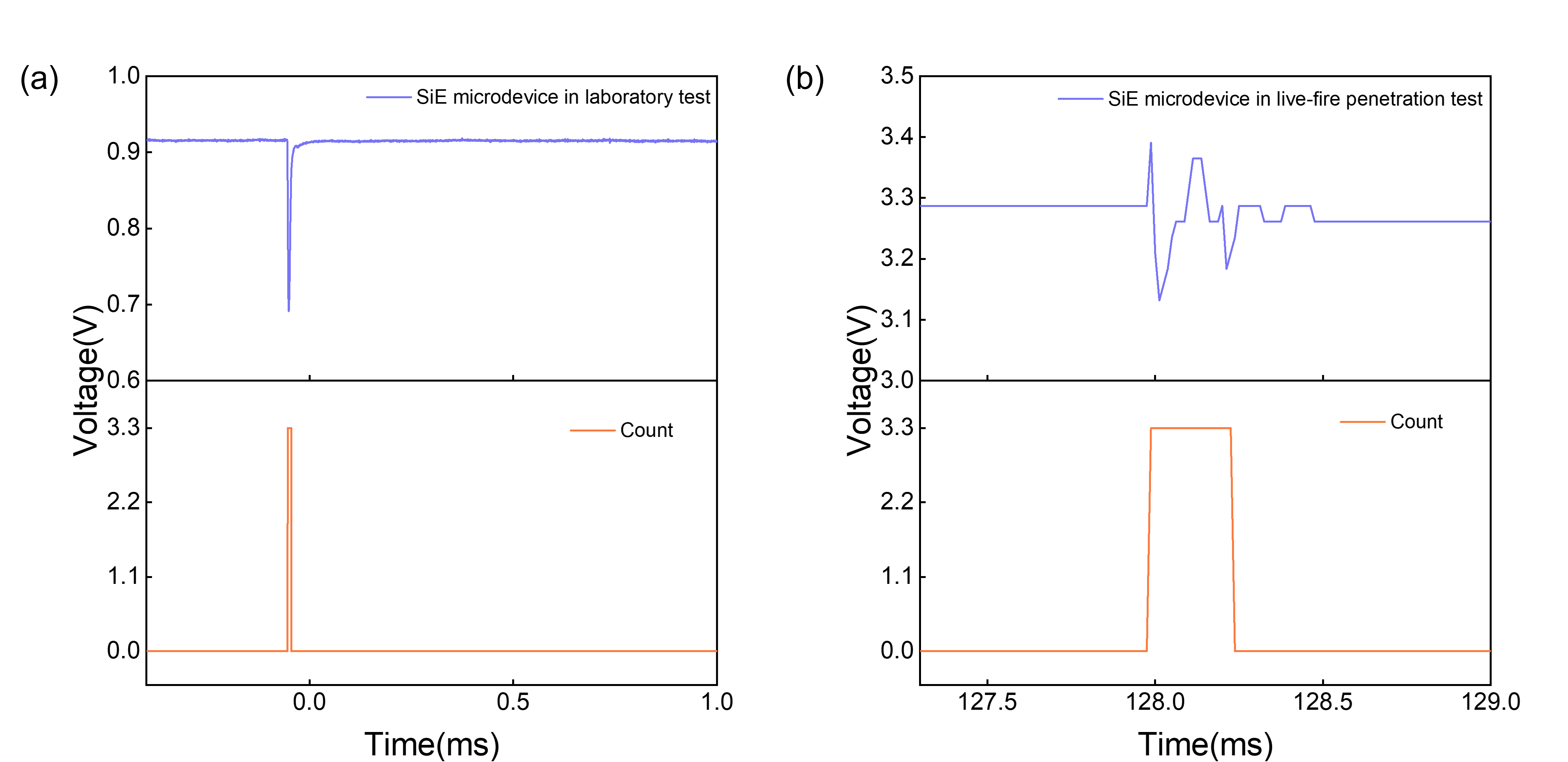


**Figure S15** Layer-counting demonstration of the SiE microdevice in laboratory and field live-fire penetration environments. (a) Simulated laboratory environment (b) Live-fire target penetration environment

Reference:

[1] Frew D J, Forrestal M J, Chen W. Pulse shaping techniques for testing brittle materials with a split Hopkinson pressure bar[J]. Experimental mechanics, 2002, 42(1): 93-106.

[2] Forrestal M J, Frew D J, Hickerson J P, et al. Penetration of concrete targets with deceleration-time measurements[J]. International journal of impact engineering, 2003, 28(5): 479-497.

[3] Zhang X, Zhao Y, Duan Z, et al. A high-g shock tester with one-level velocity amplifier[J]. Measurement Science and Technology, 2013, 24(4): 045901.

[4] Hunter S C. Energy absorbed by elastic waves during impact[J]. Journal of the Mechanics and Physics of Solids, 1957, 5(3): 162-171.

[5] Karniadakis G, Beskok A, Aluru N. Microflows and nanoflows: fundamentals and simulation[M]. New York, NY: Springer New York, 2005.

[6] Batchelor G K. An introduction to fluid dynamics[M]. Cambridge university press, 2000.

[7] Bao M. Analysis and design principles of MEMS devices[M]. Elsevier, 2005.

[8] Kogut L, Komvopoulos K. Electrical contact resistance theory for conductive rough surfaces[J]. Journal of Applied Physics, 2003, 94(5): 3153-3162.

[9] Cooper M G, Mikic B B, Yovanovich M M. Thermal contact conductance[J]. International Journal of heat and mass transfer, 1969, 12(3): 279-300.

[10] Majumder S, McGruer N E, Adams G G, et al. Study of contacts in an electrostatically actuated microswitch[J]. Sensors and Actuators A: Physical, 2001, 93(1): 19-26.

[11] Dobashi Y, Yao D, Petel Y, et al. Piezoionic mechanoreceptors: Force-induced current generation in hydrogels[J]. Science, 2022, 376(6592): 502-507.

[12] Gao D, Liu R, Yu W, et al. Gravity-induced self-charging in carbon nanotube/polymer supercapacitors[J]. The Journal of Physical Chemistry C, 2019, 123(9): 5249-5254.

[13] Chen X W, Fan S C, Li Q M. Oblique and normal perforation of concrete targets by a rigid projectile[J]. International Journal of Impact Engineering, 2004, 30(6): 617-637.

[14] Deng Y J, Chen X W, Song W J. Dynamic cavity-expansion penetration model of elastic-cracked-crushed response for reinforced-concrete targets[J]. International Journal of Impact Engineering, 2021, 157: 103981.
